# Assessment of the role of *Wolbachia* in mtDNA paraphyly and the evolution of unisexuality in *Calligrapha* (Coleoptera: Chrysomelidae)

**DOI:** 10.1002/ece3.5621

**Published:** 2019-09-07

**Authors:** Jesús Gómez‐Zurita

**Affiliations:** ^1^ Animal Biodiversity and Evolution Institute of Evolutionary Biology (CSIC‐Universitat Pompeu Fabra) Barcelona Spain

**Keywords:** cryptic species, cytoplasmic incompatibility, mtDNA, parthenogenesis, selective sweep, superinfection

## Abstract

*Calligrapha* is a New World leaf beetle genus that includes several unisexual species in northeastern North America. Each unisexual species had an independent hybrid origin involving different combinations of bisexual species. However, surprisingly, they all cluster in a single mtDNA clade and with some individuals of their parental species, which are in turn deeply polyphyletic for mtDNA. This pattern is suggestive of a selective sweep which, together with mtDNA taxonomic incongruence and occurrence of unisexuality in *Calligrapha*, led to hypothesize that *Wolbachia* might be responsible. I tested this hypothesis studying the correlation between diversity of *Wolbachia* and well‐established mtDNA lineages in >500 specimens of two bisexual species of *Calligrapha* and their derived unisexual species. *Wolbachia* appears highly prevalent (83.4%), and fifteen new supergroup‐A strains of the bacteria are characterized, belonging to three main classes: *wCallA*, occupying the whole species ranges, and *wCallB* and *wCallC*, narrowly parapatric, infecting beetles with highly divergent mtDNAs where they coexist. Most beetles (71.6%) carried double infections of *wCallA* with another sequence class. Bayesian inference of ancestral character states and association tests between bacterial diversity and the mtDNA genealogy show that each mtDNA lineage of *Calligrapha* has specific types of infection. Moreover, shifts can be explained by horizontal or vertical transfer from local populations to an expanding lineage and cytoplasmic incompatibility between *wCallB* and *wCallC* types, suggesting that the symbionts hitchhike with the host and are not responsible for selective mtDNA sweeps. Lack of evidence for sweeps and the fact that individuals in the unisexual clade are uninfected or infected by the widespread *wCallA* type indicate that *Wolbachia* does not induce unisexuality in *Calligrapha*, although they may manipulate host reproduction through cytoplasmic incompatibility.

## Graphical abstract

*Calligrapha* has several unisexual species which arose independently through interspecific hybridization of different parentals, yet they are all monophyletic for mtDNA. In this article, I test the hypothesis that this may be the result of a selective sweep induced by *Wolbachia* and that these endosymbionts may be responsible for the transition to unisexuality in these beetles. The answer to this question is that the bacteria are associated neither with the unisexual mtDNA type nor with the reproductive transition to unisexuality in *Calligrapha*, although they seem responsible for geographic patterns of mtDNA through cytoplasmic incompatibility.

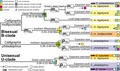

## 1 INTRODUCTION

Alphaproteobacteria of the genus *Wolbachia* (Rickettsiales: Anaplasmataceae) have been shown to live as endosymbionts of a large proportion of arthropods and also nematodes (Hilgenboecker, Hammerstein, Schlattmann, Telschow, & Werren, 2008; Weinert, Araujo, Ahmed, & Welch, 2015; Zug, Koehncke, & Hammerstein, 2012). In insects, these bacteria have been typically found or predominantly studied in germline cells (Dobson et al., 1999; Toomey, Panaram, Fast, Beatty, & Frydman, 2013; Werren, 1997), although they occur in somatic tissues of infected individuals too (e.g., Pietri, DeBruhl, & Sullivan, 2016). Their highly specialized natural history, adapted to the intracellular environment, makes their evolutionary survival often conditional on vertical inheritance from one host generation to the next, although horizontal transfer, particularly for species with intimate ecological contact and among related species, is a prevalent phenomenon as well (e.g., Heath, Butcher, Whitfield, & Hubbard, 1999; Huigens, Almeida, Boons, Luck, & Stouthamer, 2004; Russell et al., 2009). In the case of vertical transmission, it occurs via host maternal transmission through a complex interaction of the bacteria with the microtubule network of the oocytes (Ferree et al., 2005), since they are effectively removed from male gametes in late stages of spermatogenesis (Clark, Veneti, Bourtzis, & Karr, 2002). The way in which the association between the prokaryote and the eukaryotic host was established, whereby only female hosts transmit the infection, has led to one of the most Machiavellian behaviors and types of interaction of the natural world. *Wolbachia* can provide with physiological benefits for the host, such as nutritional mutualism, protection against viral infections, or even correct development of oogenesis (Dedeine et al., 2001; Hosokawa, Koga, Kikuchi, Meng, & Fukatsu, 2010; Martinez et al., 2014). Yet, they can also manipulate host reproduction and exert an influence on female reproductive fitness and sex ratio demography. As a result, they can effectively disconnect the history of mitochondrial DNA (mtDNA), also with maternal inheritance, from the history of the host. These so‐called selective sweeps have serious implications for phylogeographic and taxonomic enquiry of the host based precisely on mtDNA sequences. Moreover, the intimate association of host and endosymbiont can promote some degree of coevolution or parallel evolution, so that the confinement of the bacteria in a particular evolutionary line of the host may result in nonrandom associations of genetic diversity of both host and endosymbiont (Jiggins, Bentley, Majerus, & Hurst, 2002; Russell et al., 2009).

*Wolbachia* has evolved four strategies to attain more efficient invasive rates in their host populations (Charlat, Hurst, & Merçot, 2003; Werren, Baldo, & Clark, 2008): (a) induction of parthenogenetic reproduction (Ma & Schwander, 2017); (b) feminization of male embryos (Kageyama, Nishimura, Hoshizaki, & Ishikawa, 2002; Rousset, Bouchon, Pintureau, Juchault, & Solignac, 1992); (c) killing of male embryos (Fialho & Stevens, 2000; Hurst et al., 1999); and/or (d) sterilization of uninfected females by infected males (cytoplasmic incompatibility, also at play when different strains of the bacteria infect each parent; Engelstädter & Telschow, 2009). Because of disparate effects depending on host sexes, the potential impact of the evolutionary selfishness of these intracellular bacteria on the demography of host populations ranges from distorted sex ratios to male eradication (Werren & Beukeboom, 1998). These effects can be transitory, and “curing” the infection with antibiotics can revert them (Li, Floate, Fields, & Pang, 2014). However, they can have long‐lasting consequences, and these master manipulators have been associated, for example, with the evolutionary transition from bisexual to unisexual reproductive modes (Ma & Schwander, 2017). When the endosymbiont successfully manipulates host reproduction, a resulting side effect is that mtDNA hitchhikes with the symbiont, thus establishing a linkage disequilibrium relationship (Turelli, Hoffmann, & McKechnie, 1992). The fact that spreading of mtDNA in natural populations may be conditioned by the bacteria dynamics has dramatic consequences on the reliability of mtDNA for population, phylogeographic, and phylogenetic studies (Hurst & Jiggins, 2005). For example, mtDNA selective sweeps driven by intracellular bacteria can reduce genetic diversity, similarly to population bottleneck effects (Gompert, Forister, Fordyce, & Nice, 2008), and symbiont‐induced mtDNA introgression following interspecific hybridization may confound mtDNA‐based inference of species relationships (Galtier, Nabholz, Glémin, & Hurst, 2009; Hurst & Jiggins, 2005; Smith et al., 2012), occasionally to an exacerbated degree (Schmidt & Sperling, 2008). Similar disequilibrium effects also occur between different strains of *Wolbachia*, so that host mtDNA diversity may mirror bacterial genotypic diversity rather than true host population structure (Charlat et al., 2009; Ilinsky, 2013).

Leaf beetles in the genus *Calligrapha* are an interesting group of organisms to investigate for the presence and potential effects of reproductive manipulation by *Wolbachia*. In the first place, it is exceptional among beetles for including several examples of unisexual species, each derived from interspecific hybridization events involving different bisexual species in North America (Gómez‐Zurita & Cardoso, 2019; Gómez‐Zurita, Funk, & Vogler, 2006; Gómez‐Zurita, Vogler, & Funk, 2004; Montelongo & Gómez‐Zurita, 2015; Robertson, 1966). Moreover, it displays abnormally high levels of mtDNA paraphyly for most North American species, particularly those identified as involved in the origin of unisexual lineages (Gómez‐Zurita & Cardoso, 2019; Gómez‐Zurita et al., 2006; Montelongo & Gómez‐Zurita, 2015). Thus, bisexual species of *Calligrapha* are deeply polyphyletic for mtDNA, with individuals in at least two highly divergent clades, one with populations with normal sex ratios and named B‐clade and one exclusively comprised of females, also including representatives of the unisexual species evolutionarily derived from them, or U‐clade (Gómez‐Zurita & Cardoso, 2019; Montelongo & Gómez‐Zurita, 2015). This pattern is interpreted as each of the unisexual species of *Calligrapha* having had a history with a minimum of two waves of interspecific hybridization, with available evidence suggesting that sex was not lost immediately (Montelongo & Gómez‐Zurita, 2015). The last hybridization events leading to unisexual species occurred recently, plausibly after the Last Glacial Maximum (LGM), and they involved female‐only lineages of the otherwise bisexual parental species, suggesting that unisexuality may have actually predated the origin of unisexual species (Gómez‐Zurita & Cardoso, 2019). In this model, although hybridization appears as a necessary and important condition, this mechanism alone is not sufficient to explain the origin of unisexuality.

Given that a particular type of ancestral mtDNA is associated with unisexuality in *Calligrapha*, this is suggestive that the same process that introgressed this specific mtDNA could have simultaneously transferred a factor that would eventually lead to the evolution of unisexuality. There is no satisfactory explanation for this idea. However, among the possible hypotheses worth testing, the most prominent, linking both the origins of unisexuality and also selective sweeps of mtDNA, advocates for the potential role of intracellular symbionts in the process (Montelongo & Gómez‐Zurita, 2015). As highlighted above, the involvement of these endosymbionts in the evolution of *Calligrapha* and important life‐history traits, such as reproductive mode, could additionally explain other observations in this system, such as the highly distorted sex ratios in some populations (Brown, 1945; Robertson, 1966) or evolutionary lineages (Gómez‐Zurita & Cardoso, 2019), and extensive species mtDNA polyphyly (Gómez‐Zurita et al., 2006; Montelongo & Gómez‐Zurita, 2015), among others.

In order to test the hypothesis that *Wolbachia* may be responsible for these observations, this work shows a replicated study in several species of *Calligrapha* for which there is a detailed understanding of their genetic diversity and phylogeographic processes (Gómez‐Zurita & Cardoso, 2019). The study will try to establish the association between their mtDNA diversity, their geographic distribution, and the apparent conflict with their taxonomic boundaries with the presence of *Wolbachia*. This will be accomplished after screening first the same samples studied by Gómez‐Zurita and Cardoso (2019) for the presence of *Wolbachia*, which has not been attempted before in the genus, and characterizing their strains. Based on this information, the main objective of the study will be testing for links between *Wolbachia* and unisexuality in *Calligrapha*, and more generally investigating if and how *Wolbachia* may have affected mtDNA diversity in this host.

## 2 MATERIAL AND METHODS

### 2.1 Sampling and major phylogeographic lineages of *Calligrapha*

**Figure 1:**
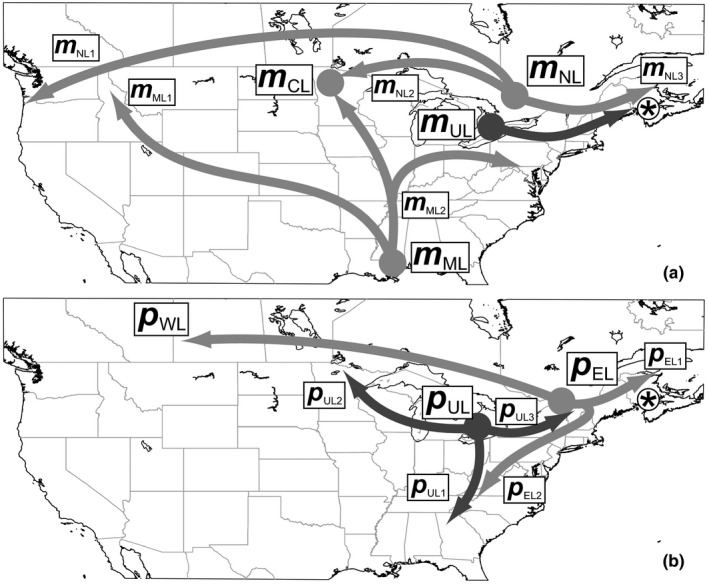
Schematic summary of phylogeographic lineages of *Calligrapha* considered in this work and based on the results of Gómez‐Zurita and Cardoso (2019), including (a) four main lineages (*m*_ML_, *m*_CL_, *m*_NL_, and *m*_UL_) in *C. multipunctata* and (b) three main lineages (*p*_WL_, *p*_EL_, and *p*_UL_) in *C. philadelphica*. The *m*_UL_ and *p*_UL_ lineages also include the unisexual species *C. suturella* and *C. vicina*, respectively. The asterisk marks the hypothetical area where *m*_NL3_ and *p*_EL1_ lineages met and hybridized, establishing a population of *C. philadelphica* with typical *C. multipunctata* mtDNA

This study used the specimens, DNA, and *cox1* and *Wg* sequence data previously analyzed by Gómez‐Zurita and Cardoso (2019). The sample included representatives of two bisexual species of *Calligrapha* with transcontinental ranges in North America, *C. philadelphica*, and two subspecies of *C. multipunctata*, as well as two unisexual species, *C. vicina* and *C. suturella*, evolutionarily derived from the unisexual populations of each of the previous two, respectively (Montelongo & Gómez‐Zurita, 2015). A total of 506 specimens were studied, including 322 individuals of *C. multipunctata bigsbyana*, 22 of *C. multipunctata s. str*., 148 of *C. philadelphica*, and seven each of *C. suturella* and *C. vicina* (Table S1). MtDNA and nuclear *Wg* data were consistent in identifying seven major and 13 geographically concordant evolutionary lineages in the sample (Gómez‐Zurita & Cardoso, 2019). In bisexual *C. multipunctata*, four major lineages were distinguished (Figure 1a): (a) evolutionary branch associated with the Mississippi basin (*m*_ML_) with one lineage expanding northwest, to establish populations in the Rocky Mountains (*m*_ML1_), and one expanding north to the Northern Great Plains in the Upper Mississippi and to the Alleghany region along the Ohio River Basin (*m*_ML2_); (b) branch established and expanding in the Northern Great Plains (*m*_CL_); (c) northern branch (*m*_NL_) with three main lineages, one reaching the Pacific Northwest (*m*_NL1_), one established in the Northern Great Plains (*m*_NL2_), and one in New England and the northern Atlantic regions (*m*_NL3_), the latter hybridizing with *C. philadelphica* in its easternmost front of expansion in the Holocene; and (d) unisexual lineage of *C. multipunctata* (*m*_UL_), expanding from the Great Lakes region, giving rise to *C. suturella* upon hybridization with *C. ignota* after the LGM (Montelongo & Gómez‐Zurita, 2015). In *C. philadelphica*, three major lineages were present (Figure 1b): (a) western lineage, west of the Saint Lawrence River and reaching southeastern plains of Alberta (*p*_WL_); (b) eastern lineage, east of the Saint Lawrence River (*p*_EL_), with one lineage expanding northeast (*p*_EL1_) and one expanding east and southwest (*p*_EL2_) along the western Appalachian slopes; and (c) unisexual lineage of *C. philadelphica* (*p*_UL_), with independent branches expanding south to Georgia along central and southern Appalachians (*p*_UL1_), west to Minnesota (*p*_UL2_), and east to New England (*p*_UL3_), the latter giving rise to *C. vicina* via hybridization with ancestral populations of *C. rowena* after the LGM (Montelongo & Gómez‐Zurita, 2015).

### 2.2 MLST genotyping of *Wolbachia*

Each individual DNA extraction of *Calligrapha* was used as template to investigate the presence and strain of *Wolbachia* infecting every beetle and using the multilocus sequence typing system (MLST) designed for *Wolbachia* by Baldo et al. (2006). We used the same loci (*gatB*, *coxA*, *hcpA*, *fbpA*, and *ftsZ*) and PCR amplification protocols of Baldo et al. (2006), which worked well in our hands, except for the *ftsZ* locus, which was amplified in most cases using combinations of nondegenerate custom primers that amplified fragments between 522 and 624 bp and encompassing the whole region of interest: ftsZf2 (5′‐GTCTTGGTGCTGGTGCTTTG) or ftsZf3 (5′‐GGTGCTTTGCCTGATGTTGG) with ftsZr2 (5′‐TGGATATTGCAGCTTCCGCA) or ftsZr3 (5′‐TGGCTGCAGCATCAACTTCA). PCR products were purified with ammonium acetate 5M and isopropanol and sequenced in both directions using the same PCR primers and the Sanger method with BigDye Terminator 3.1 technology and capillary sequencing on a 3730xl DNA Analyzer (Applied Biosystems). Sequences were trimmed to their respective MLST fragment size (Baldo et al., 2006), aligned using the L‐INS‐i algorithm of MAFFT 7 (Katoh & Standley, 2013), and filtered to allele sequence data using analytical tools in Geneious R9 (Biomatters Ltd.). Each allele was identified with reference to the curated Wolbachia MLST Database (update: 17 April 2017) using the associated PubMLST online public resource (https://pubmlst.org/wolbachia/; Jolley & Maiden, 2010), but since the curation of *Wolbachia* data was very unfortunately discontinued (J. Werren, personal communication), the identity of the obtained alleles was additionally checked against the latest release of GenBank too (31 July 2018). A representative allele of each of these sequences was deposited in GenBank, with sequence accession numbers LR135794–LR135810.

### 2.3 Association test between mtDNA and *Wolbachia* diversity: contingency tests

Gómez‐Zurita and Cardoso (2019) identified a number of mtDNA lineages in the species of *Calligrapha* under study, their distribution, and the history of their range changes based on the genealogy of *cox1* and a major association of these lineages with different allelic and genotypic characteristics of *Wg* (Figure 1). These lineages were established in part based on an unrooted *cox1* genealogy obtained under statistical parsimony and a nested‐clade design resulting from the implementation of Templeton, Boerwinkle, and Sing (1987) algorithm. For visualization purposes in the current study, the same nested clades of Gómez‐Zurita and Cardoso (2019) were mapped on the phylogeny of *cox1* haplotypes obtained under maximum likelihood with RAxML 7.2.8 (Stamatakis, 2006), specifying a GTR+G+I evolutionary model and 10 replicates of random addition of taxa, and assessing node support based on 100 bootstrap pseudoreplicates. The association between the occurrence and type of *Wolbachia* infections and mtDNA evolutionary diversity in *Calligrapha*, as well as a possible scenario for the history of this association by reference to the phylogeographic inferences of Gómez‐Zurita and Cardoso (2019), was explored using permutation contingency tests of discrete variables. To carry out this analysis, the nested clades of the *cox1* haplotype statistical parsimony genealogy of Gómez‐Zurita and Cardoso (2019), retaining here the same numbering as in that work for easier cross‐reference, were transformed into a categorical variable to test the association of these groups of related haplotypes with different *Wolbachia* genotypes. In turn, *Wolbachia* infections were transformed into three types of categorical variables: infection status and type of infection (uninfected vs. single infection vs. double infection); genotype group (one of three main groups); or specific genotype. Permutation chi‐square exact contingency tests of these variables on the structure defined by the *cox1* genealogy, best known as nested‐clade analysis (Templeton & Sing, 1993), were carried out for all contingency matrices where both variables showed observations. The tests and their significance were carried out using 9,999 data permutations for each contingency matrix using the function perm.ind.test of the R package wPerm 1.0.1 (Weiss, 2015). Permutation of the contingency matrices simulated the null hypothesis of no association between mtDNA genetic differences and the type and characteristics of the infection with *Wolbachia*, and a significant result was interpreted as the existence of statistical differences between the infections of tested mtDNA groups.

When a significant association was obtained in the previous tests, the diversity of *Wolbachia* was tested also for an association with the *Wg* genotypes of the same individuals involved in the original test to find whether the association was specific for mtDNA or if there was signal of association with the nuclear marker as well.

### 2.4 Association test between mtDNA and *Wolbachia* diversity: ancestral states

Bayesian inference of ancestral states (Ronquist, 2004) was used as a complementary tool to investigate the association of specific mtDNA evolutionary lineages in *Calligrapha* and the type of *Wolbachia* they carried. Bayesian trait analysis and ancestral character inference were carried out with BEAST 1.8.4 (Drummond, Suchard, Xie, & Rambaut, 2012) on a reduced matrix where each different combination of taxon, *cox1* haplotype, and associated *Wolbachia* sequence type(s) was considered. This matrix was analyzed under an HKY+I+G model and a relaxed lognormal clock. An additional partition was created to represent *Wolbachia* sequence type traits associated with each species/haplotype and allowing for ambiguous codes to account for double infections. Two coding schemes were used: one considering each ST or ST combination as well as the uninfected state (31 states), and one grouping sequence types in three major groups as recognized in this study (four states). This trait was allowed to change under a symmetric substitution model and a random local clock, independent from the nucleotide sequence evolution clock. Both nucleotide sequence and infection partitions were linked to a single tree estimated under a constant‐size coalescent model. Infection states for all ancestors, including the root of the tree, were deduced and annotated on the obtained tree topologies. Tree searches were based on two independent MCMC chains of 70 million generations each, sampling one every 7,000 trees, and the results of the search were analyzed with Tracer 1.6.0 (Rambaut, Suchard, Xie, & Drummond, 2014) and TreeAnnotator 1.8.4 (Drummond et al., 2012), excluding 10% of the initial, stabilizing phase, and visualized with FigTree 1.4.3 (available from http://tree.bio.ed.ac.uk/software/figtree/).

## 3 RESULTS

### 3.1 Incidence of infection and strains of *Wolbachia*

Most samples of *Calligrapha* (83.4%) were confirmed for infection with *Wolbachia* by consistent amplification of MLST markers, and 71.6% of them showed evidence of double infections, presenting double sequence peaks for all markers. The availability of clean allele sequences for each gene in numerous specimens and the expected double‐peak patterns of their combinations allowed to separate easily the alleles of coinfecting strains in all but one instance of *gatB*, found in one individual of *C. multipunctata bigsbyana* from Quyon (Quebec). A number of samples (9.1%), upon several PCR trials, failed to amplify any of the five MLST markers of *Wolbachia*, indicating the absence of infection, an idea reinforced by their trend to affect coherent sets of samples (e.g., sharing a certain *cox1* haplotype, coming from the same locality or belonging to the same evolutionary lineage). Some samples produced faint bands for one (7.3%) or two (0.2%) of the loci tested, but inconsistently and only in some of the repeated trials. Nonetheless, we also tried to sequence these PCR products, always producing low‐quality sequences and at most for one of the strands. These results could represent idiosyncrasies of PCR or template DNA quality issues, but their inconsistency and unrepeatability were suggestive of PCR artifacts or contamination surfacing when forcing PCR conditions.

**Figure 2:**
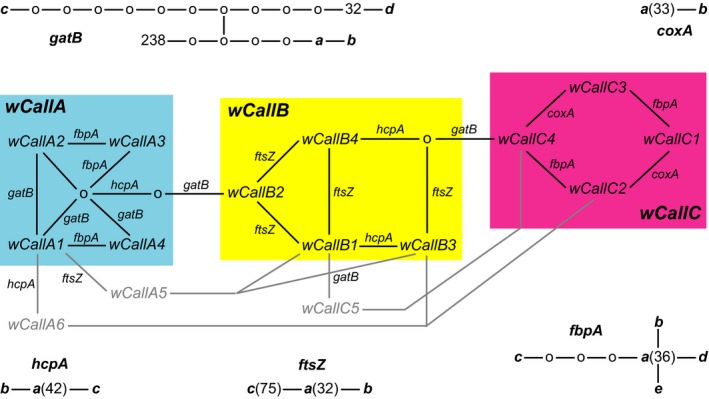
Intralocus and MLST evolutionary relationships of *Wolbachia* characterized in the sample of four species of *Calligrapha*. Relationships among alleles of individual MLST loci (identified with the codes given in Table S2) and among *wCall* sequence types are represented using statistical parsimony networks where edges identify individual mutations in the former and allele changes in the latter. Three main groups of *wCall* MLSTs are identified based on changes occurring in two or more individual loci

In total, the sample of *Wolbachia* yielded four *gatB*, two *coxA*, three *hcpA*, three *ftsZ*, and five *fbpA* alleles, of which only one allele each of *coxA*, *hcpA*, and *fbpA* and two of *ftsZ* had been described previously (Table S2). Previously uncharacterized alleles were evolutionarily close to already known variants (Figure 2; Table S2). These 18 alleles produced 15 genotypes or sequence types (ST) belonging to three main groups, called here *wCallA* to *wCallC*, mostly defined by the *gatB* allele, with all genotypes from one group separated by mutations in two or more MLST loci from genotypes in other such group (Table 1; Figure 2). Half of these genotypes were recognized directly from singly infected individuals. In turn, deduced genotypes differed from one of the former in just one of the five MLST loci (Table 1), making their separation straightforward by discounting the state of the corresponding observed genotype from the polymorphism established empirically (Table 2). All the alleles and their combinations could be referred to the supergroup A of *Wolbachia*.

**Table 1:** *Wolbachia* sequence types (ST) observed in the sample of *Calligrapha* in the case of single infections or deduced from individuals with double infections

*Wolbachia* ST	Alleles^a^	*N*	*Calligrapha* cox1 haplotypes or coinfecting ST
Uninfected	–	83	11B1, 1B6, 2B14, 1B15, 2B18, 1**B27**, 4**B28**, 1**B29**, 2B31, 1B32, 2B33, 1B35, 5B46, 3B47, 1B48, 3B57, 2B58, 3**B59**, 1**B69**, 1**B73**, 9**U1**, 1**U2**, 2**U3**, 4***U3***, 1**U4**, 6U5, 4***U5***, 1U7, 6U8, 1**U13**
Observed			
*wCallA1*	***a***,33,42,32,36	61	13B1, 1B12, 2B18, 1B38, 1B45, 1B46, 16B49, 1B54, 3**B63**, 6**U1**, 2***U3***, 1***U5***, 4**U9**, 2**U10**, 4**U11**, 2**U12**, 1**U13**
*wCallA2*	***b***,33,42,32,36	27	3**B1**, 1**B7**, 23B14
*wCallA3*	***b***,33,42,32,***d***	3	1B39, 1B40, 1B41
*wCallA4*	***a***,33,42,32,***e***	1	1B1
*wCallB1*	***c***,33,***c***,***b***,***b***	16	2B14, 8*B33*, 2*B34*, 2*B35*, 2*B36*
*wCallB2*	***c***,33,***c***,32,***b***	6	1*B33*, 2*B34*, 2*B35*, 1*B56*
*wCallC2*	***d***,33,***b***,75,***c***	6	3**B59**, 3**B68**
Deduced			
*wCallA5*	***a***,33,42,***b***,36	–	*wCallB4, wCallC1*
*wCallA6*	***a***,33,***b***,32,36	–	*wCallA1, wCallC2*
*wCallB3*	***c***,33,***b***,***b***,***b***	–	*wCallA1*
*wCallB4*	***c***,33,***c***,75,***b***	–	*wCallA1, wCallA5*
*wCallC1*	***d***,***b***,***b***,75,***c***	–	*wCallA1, wCallA4, wCallA5*
*wCallC3*	***d***,***b***,***b***,75,***b***	–	*wCallA1*
*wCallC4*	***d***,33,***b***,75,***b***	–	*wCallA1*
*wCallC5*	***d***,33,***c***,***b***,***b***	–	*wCallA1*

Note

In the case of the latter, coinfecting STs are listed. In the case of the former and uninfected individuals, the *cox1* haplotypes and the frequency of beetle individuals where the *Wolbachia* ST is found are given, with an indication of species identity (round font: *C. multipunctata bigsbyana*; italics: *C. multipunctata s. str*.; bold: *C. philadelphica*; bold italics: *C. suturella* and *C. vicina*).

a Characteristics of alleles and their repositories described in Table S2.

**Table 2:** Double *Wolbachia* infections in the sample of *Calligrapha*, identifying coinfecting MLST types, their frequency, and the *cox1* haplotypes of affected individuals and their proportion in the sample (their taxonomic identity is coded as in Table 1)

ST1	ST2	Freq.	*Calligrapha* cox1 haplotypes
*wCallA1*	*wCallA6*	1	1**B63**
*wCallA1*	*wCallB1*	99	1**B1**, 1**B7**, 1B8, 4B9, 1B10, 4B11, 1B13, 2B16, 10B24, 1B25, 1**B26**, 13**B29**, 1B37, 8B42, 1B44, 25B49, 2B50, 1B51, 1B52, 10B53, 1B55, 1**B63**, 1**B64**, 6**U1**, 1***U3***
*wCallA1*	*wCallB3*	1	1B49
*wCallA1*	*wCallB4*	1	1**B29**
*wCallA1*	*wCallC1*	116	68B1, 1B2, 1B3, 1B5, 40B18, 1B19, 1B20, 1B21, 1***U5***, 1***U6***
*wCallA1*	*wCallC2*	27	15**B59**, 1**B60**, 1**B61**, 3**B62**, 3**B65**, 1**B66**, 1**B71**, 1**B72**, 1**U1**
*wCallA1*	*wCallC3*	3	2B1, 1B18
*wCallA1*	*wCallC4*	1	1**B65**
*wCallA1*	*wCallC5*	1	1**B4**
*wCallA2*	*wCallB1*	36	4**B1**, 1**B7**, 6B14, 5B17, 1**B22**, 1**B23**, 2**B26**, 7**B27**, 7**B28**, 2**B30**
*wCallA2*	*wCallC2*	4	4**B71**
*wCallA4*	*wCallC1*	1	1B1
*wCallA5*	*wCallB4*	3	1**B29**, 2**U1**
*wCallA5*	*wCallC1*	1	1B1
*wCallA6*	*wCallC2*	4	3**B67**, 1**B70**
*wCallA1*	*wCallB1r*	3	2B42, 1B43
*wCallB1* a	*wCallC1* a	1	1B18

a *wCall* STs could not be determined because of uncertainty in establishing the *gatB* alleles. This is the only individual in the sample where strains *wCallB* and *wCallC* coexist, apparently. *wCallB1r* shows an abnormal *gatB* sequence which could be interpreted as a mosaic between ***c*** and ***d*** alleles.

There were few cases of intralocus and interlocus recombinants in the genetic sample of *Wolbachia*. The former were represented by three specimens of *Calligrapha multipunctata bigsbyana* from the Grass Island County (New York), where the *gatB* locus was seemingly a chimera between “c” and “d” alleles, whereby “c” is also present in the population (Table 2). Moreover, up to three rare STs (*wCallA5*, *wCallA6*, and *wCallC5*), observed in ten individuals, could be proposed of recombinant origin between loci, as they were responsible for loops in an ST evolutionary network connecting at least two divergent STs (Figure 2). Most double infections detected in *Calligrapha* always involved one of the *wCallA* STs with either a *wCallB* or a *wCallC* strain of *Wolbachia*. The only exceptions were a single case of double infection inferred between two strains of group *wCallA* in one individual of *C. philadelphica* from Pinchot Lake in York County (New York), and an uncertain ST because of the ambiguous resolution of *gatB* polymorphisms mentioned above and seemingly involving *wCallB* and *wCallC* variants, both present in the same population (Table 2).

### 3.2 Geographic ranges and prevalence of *Wolbachia* strains

The most prevalent *Wolbachia* ST in *Calligrapha* was *wCallA1*, infecting alone or in combination with other strains up to 74.2% of individuals, including representatives of all the species analyzed, except *C. multipunctata* s. str. Correspondingly, this type of *Wolbachia* appeared widely distributed throughout most of the geographic range considered here, in the eastern half of North America, from Saskatchewan to Nova Scotia and south to NE Texas, Georgia, and Alabama (Figure S1a). The next most abundant *wCallA* type in the sample was *wCallA2*, infecting 15.8% of specimens, including *C. philadelphica* and a genetically coherent group of *C. m. bigsbyana*, and it was distributed in the northern periphery of the studied range, in isolated populations along the Pacific coast, southern Alberta, and Maine and New Brunswick (Figure S1b). The second most abundant ST, either in single or in double infections of all species but *C. suturella*, was *wCallB1*, and it was present in 35.7% of infected individuals, also occupying most of the studied range across the continent, including the interior mountain beetle populations in Oregon and Utah, those in the upper Mississippi, the James and Missouri rivers, and as far south as the localities studied in western North Carolina (Figure S1c). All other *wCallB* types were much less prevalent and occupying narrower taxonomic and geographic areas within this same range, always found in localities where *wCallB1* was present (Figure S1c). *wCallC1* was very abundant, present in 27.9% of individuals analyzed, but exclusively found doubly infecting *C. m. bigsbyana* and *C. suturella* from eastern Ontario to Nova Scotia and south to Massachusetts, with one isolated occurrence in the northern Michigan peninsula (Figure S1d). Finally, *wCallC2* was the fifth in prevalence in the sample (9.7% of infected *Calligrapha*), and it was only found in *C. philadelphica* from nearly the same area as *wCallC1*, from the Lake Ontario (southernmost locality) to New Brunswick and the Gaspe Peninsula (Figure S1e). In fact, the *wCallC* type was very coherent geographically, occupying an area from the Great Lakes area to Nova Scotia (Figure S2). Unisexual species of *Calligrapha* were either uninfected (57.1%) or infected with the *wCallA1* type, solely (21.4%) or simultaneously with *wCallC1* (14.3%, only *C. suturella*) or *wCallB1* (7.1%, only *C. vicina*).

Individuals with single and double infections, as well as lacking any sign of infection, could be found nearly throughout the whole studied range (Figure S3). However, doubly infected individuals were absent in samples captured in the Rockies and in southern populations of the studied range, with the southernmost double infections detected in western North Carolina (Figure S3). The individuals in these southern or mountainous areas were either uninfected or singly infected by *wCallA* or *wCallB Wolbachia*. Other uninfected individuals were found occasionally within infected populations (Figure S3a).

### 3.3 Association of genetic diversity of *Calligrapha* and *Wolbachia*

**Figure 3:**
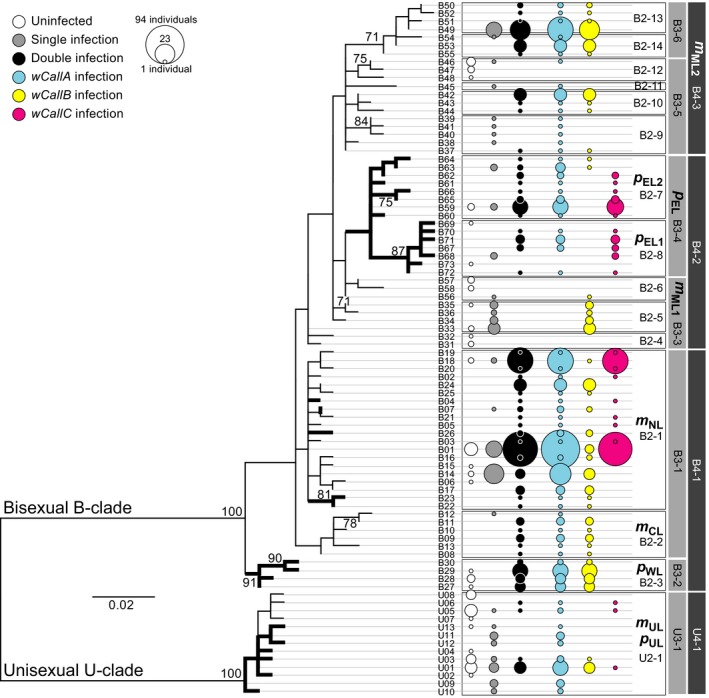
Maximum‐likelihood tree (likelihood score = −1,869.796071) of *cox1* bisexual (B) and unisexual (U) haplotypes of *Calligrapha multipunctata* s.l. (thinner branches) and *C. philadelphica* (thicker branches). Bootstrap support > 70% is shown next to the corresponding node. Haplotype groups consistent with phylogeographic lineages of Figure 1 and with the 2‐ and 3‐level nested clades of Gómez‐Zurita and Cardoso (2019), retained here for easier cross‐referencing, are shown on the right panel. Bubble graphs show the proportion of individuals with a given haplotype which are uninfected, infected by a single strain of *Wolbachia*, with double infections, and infected by *Wolbachia* of sequence types *wCallA*, *wCallB*, and *wCallC*

The maximum‐likelihood tree of *cox1* haplotype data was consistent with the multifurcate unrooted topology of Gómez‐Zurita and Cardoso (2019), with very few supported clades beyond the clear separation of bisexual and unisexual groups (B‐ and U‐clades, respectively; Figure 3). Figure 3 also shows the frequency, type, and infecting strains of *Wolbachia* characterized for individuals with each haplotype, as well as the arrangement of these haplotypes in relatedness groups mapped on the maximum‐likelihood tree. A number of nonrandom patterns emerged from the visual inspection of this tree: (a) Infection with *Wolbachia* was widespread in *Calligrapha* and typically as double infections, with *wCallA* being dominant in this pattern; (b) some groups of related haplotypes, including *m*_ML1_ (B2‐4, B2‐5, B2‐6), part of *m*_ML2_ (B2‐9, B2‐11, B2‐12), and to a good extent the unisexual lineages *m*_UL_ and *p*_UL_ (U2‐1), showed an alternative pattern, with dominance of uninfected individuals or infected by a single type of *Wolbachia*; and (c) *wCallC*‐type infections appeared restricted to haplotypes in the *m*_NL3_ (B2‐1) and *p*_EL_ (B2‐7, B2‐8) lineages, and marginally in the unisexual U2‐1 group (e.g., *m*_UL_), usually contributing the majority of double infections with *wCallA* bacteria.

The existence of those and other more subtle nonrandom patterns was also confirmed statistically on the evolutionary lineages of *Calligrapha* defined by the nested design of Gómez‐Zurita and Cardoso (2019). Permutation contingency tests of haplotype groups and different ways to code the genetic diversity of infecting *Wolbachia* showed statistically significant associations at all higher hierarchical levels (whole dataset, 5‐ and 4‐level clades) in the mtDNA genealogy, and progressively fewer significant associations at lower levels: 83.0% of 3‐level, 31.0% of 2‐level, and 18.5% of 1‐level clades. These associations were generally found for all coding strategies of infection, including its presence/absence, whether it was unique or by two strains of *Wolbachia*, but also considering the main groups of *Wolbachia* (*wCallA–wCallC*), and the specific sequence types associated with each *cox1* haplotype group or *Wg* genotypes (Table 3). Of particular interest was the statistically significant difference found between bisexual and unisexual mtDNA lineages, the latter characterized by the lack of infection or by single infections by *wCallA Wolbachia*, omnipresent in the system. This result alone, where the shift to unisexuality appears associated with the loss, not the gain, of an infection, makes it very unlikely that the endosymbiont was responsible for the shift in reproductive mode in *Calligrapha*.

**Table 3:** Results of permutation contingency tests between categorical variables defined by the nested groups within each higher‐level nesting category as defined in Gómez‐Zurita and Cardoso (2019) and shown in Figure 3, and different partitioning of the characteristics of *Wolbachia* infections

Clade	*N*	Infection level	Infection level and group	Group	Genotype^a^	Genotype	Infection level and genotype^a^	Infection level and genotype	*N*	Infection level and *Wg* genotype	*N*	*Wolbachia* and *Wg* genotypes
B1‐1	114	10.5062ns	26.3025ns	33.9207ns	152.1504*	135.5373*	149.6708ns	131.9611ns	99	72.3118ns	92	417.9370*
B1‐3	41	30.9013***	30.9013**	21.9500*	49.5733***	30.1257***	69.0863***	51.9296***	38	26.4478ns	37	49.7538ns
B1‐12	18	0.1324ns	0.1324ns	0.1328ns	17.8763*	17.3022*	18.0000*	17.0000*	18	2.1176ns	17	19.0269ns
B1‐20	9	3.6000ns	12.6000*	8.0357ns	8.7500ns	NA	12.6000*	NA	9	3.7500ns	9	15.2500ns
B1‐23	13	23.0000**	23.0000**	24.7500*	35.7500*	13.1250ns	39.0000***	24.0000**	13	18.2963ns	12	23.6667ns
B2‐1	220	61.4040***	188.9107***	125.3078***	292.1368***	285.1533***	345.3811***	325.4300***	196	164.0585ns	229	940.5087*
B2‐3	37	2.9321ns	2.9321ns	3.1912ns	28.6671***	25.6945***	26.6591***	23.9338***	36	8.0000ns	30	32.3180ns
B2‐7	43	4.1658ns	30.9205**	14.5367*	23.7644**	21.3997**	37.4397***	34.0385***	37	21.3215ns	34	63.8681ns
B3‐1	232	2.4728ns	40.2968***	32.8445***	36.7553**	34.1012**	79.9876***	73.1354***	207	170.0170ns	240	961.3259*
B3‐3	31	16.0423***	16.0423***	16.0423***	16.8095**	2.4561ns	16.8095**	2.4561ns	28	19.8333*	20	12.6191ns
B3‐4	58	0.7942ns	2.7529ns	1.3826ns	27.4388***	27.4371***	30.3779***	30.8729***	52	20.0018ns	47	120.6781ns
B3‐5	28	43.5436***	43.5436***	39.6610***	55.7508***	16.5128**	52.4103***	24.1641***	26	22.2897ns	19	23.4069ns
B4‐1	269	10.4523**	73.6027***	60.9202***	76.1527***	76.4096***	82.1639***	85.1756***	243	190.9150ns	270	1,187.9560*
B4‐2	89	36.0953***	73.8563***	98.5158***	98.8854***	99.1529***	73.8563***	73.0000***	80	89.1380**	67	281.3043ns
B4‐3	86	20.8517***	20.8517***	23.2303***	32.0679***	9.9204*	29.7042***	10.4934*	84	55.4334ns	77	87.0427ns
B5‐1	444	29.7130***	233.0242***	85.6091***	443.6518***	451.1974***	515.8262***	529.3976***	407	280.4885**	415	2,768.9090*
U2‐1	69	37.5108***	42.9322***	33.2761***	39.6440*	26.53214*	46.5465***	33.6566**	62	48.0701**	31	73.3656*
Total	506	82.6303***	96.0976***	118.8766***	136.1692***	46.55018**	129.9034***	97.6395***	469	370.2899***	446	2,834.5360*

Note

“Infection level” considers single, double, and lack of infection; “group,” any of *wCallA* to *wCallC* types; and “genotype,” individual MLST types. Only nesting categories with any of the tests producing significant results are shown. The last four columns show the results of permutation contingency tests of *Wolbachia* infection type and genotypes relative to the *Wg* genotypes of the individuals involved as characterized by Gómez‐Zurita and Cardoso (2019). In every case, *N* represents the number of individual observations considered in the test.

a The test includes a category for uninfected individuals.

* .05 ≥ *p* > .01

** .01 ≥ *p* > .001

*** *p* ≤ .001 (Significance).

### 3.4 Temporal buildup of the association of *Calligrapha* with *Wolbachia*

**Figure 4:**
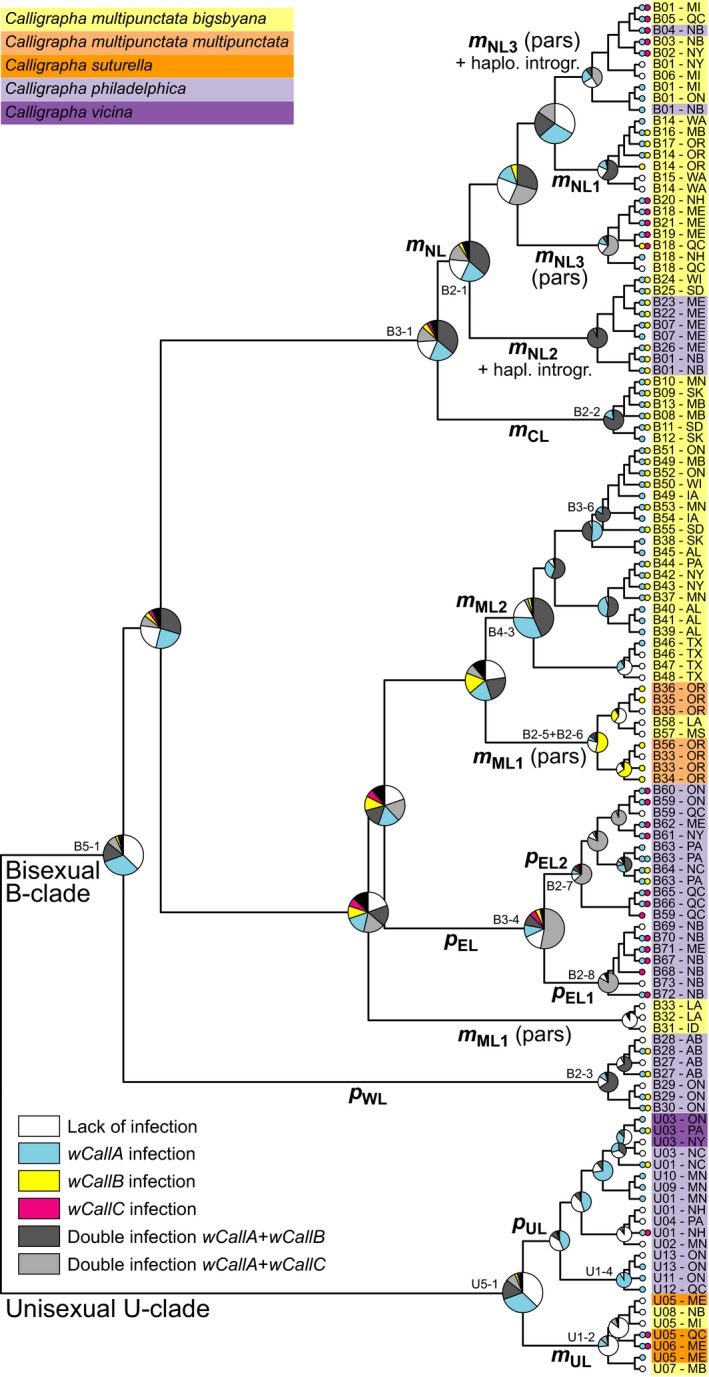
Evolution of the level and type of *Wolbachia* infection as deduced from a Bayesian trait analysis of *Wolbachia* associations on the *cox1* genealogy of *Calligrapha*. The specific infection status of each terminal in the genealogy is shown with colored symbols using the same code as in the legend, and pie charts at each node show the relative posterior probabilities for each type of infection inferred for a particular ancestor. The major phylogeographic lineages from Figure 1 that can be identified in the genealogy are labeled accordingly, as well as their correspondence with the nested clades of Gómez‐Zurita and Cardoso (2019)

The close association between mtDNA genealogy (and *Wg* diversity to some extent) and *Wolbachia* infections showed that it was possible to estimate a dominant ancestral infection state for major groups of haplotypes, strengthening the perception of a tightly linked association between the evolution of mtDNA in *Calligrapha* and their endosymbionts (Figure 4). For most major lineages, their ancestor was inferred as carrying symbionts compatible with their extant infection status, or in other words, closely related haplotypes typically showed similar infection status. For example, the most recent common ancestor of haplotypes B59‐B73 of *C. philadelphica* (*p*_EL_ lineage; clade B3‐4 of Gómez‐Zurita & Cardoso, 2019) was inferred with 51.6% probability of carrying a double *Wolbachia* infection of the *wCallA* and *wCallC* types, as found in 63.8% of the individuals examined (the next possible state inferred was 14.7% probability of lack of infection). Also, the ancestor of the large clade including the *m*_CL_ and *m*_NL_ lineages (clade B3‐1) was inferred a 56.7% combined probability of carrying a *wCallA*‐type *Wolbachia* alone or, most likely, doubly infecting with *wCallB*‐type *Wolbachia*. The most recent common ancestor of the U‐clade showed a highest probability of being either uninfected (37.7%) or infected by a *wCallA*‐type *Wolbachia* (32.5%), reinforcing the idea of the lack of association of unisexuality in *Calligrapha* with any particular type of *Wolbachia* infection.

## 4 DISCUSSION

### 4.1 *Calligrapha* beetles host a diverse and partially incompatible array of *Wolbachia*

The first relevant, novel result from this study is confirming that *Wolbachia* occurs with high prevalence in natural populations of *Calligrapha* beetles. Fifteen closely related supergroup‐A MLST variants of *Wolbachia* (Lo, Casiraghi, Salati, Bazzocchi, & Bandi, 2002) were characterized in a continental sample of four species of *Calligrapha*. None of these variants had been previously recorded in other insect hosts, although similar STs, sharing allele combinations for up to four MLST markers, had been described already for other beetles. These include variants *Cobs_A* in the weevil *Ceutorhynchus obstrictus* (Floate, Coghlin, & Dosdall, 2011) and *Ocac_A* in the leaf beetle *Oreina cacaliae* (Montagna et al., 2014). The diversity of *Wolbachia* associated with *Calligrapha* is relatively low and exclusive, suggesting limited divergence within the host. However, the relative conservation of MLST loci implies a certain risk to misjudge horizontal transfer between distant *Calligrapha* species or from other hosts (e.g., Kraaijeveld, Franco, Knijff, Stouthamer, & Alphen, 2011). The successful recognition of meaningful evolutionary and geographic patterns in the data is interpreted here as limited or absent random horizontal transfer events, which would obscure these patterns.

Another relevant finding recognizes that most *Calligrapha* specimens studied here support double infections of *Wolbachia* and that coexisting strains are not random, but consist of simultaneous infections of *wCallA* with either *wCallB* or *wCallC* bacteria. Very early, since the pioneering screenings of invertebrates for the presence of *Wolbachia* (Perrot‐Minnot, Guo, & Werren, 1996), it became apparent that the host could support two—and in some cases up to three, as in the adzuki bean beetle (Kondo, Ijichi, Shimada, & Fukatsu, 2002)—strains of the endosymbiont in their tissues. Double infections, maintained in certain circumstances by offering a selective advantage to both symbionts and the host (Engelstädter, Hammerstein, & Hurst, 2007; Vautrin, Charles, Genieys, & Vavre, 2007), were soon interpreted under the light of the effects on host phenotype as well as the compatibility dynamics between bacterial strains affecting the outcome of host reproduction. These effects of superinfections range from feminization of males (Hiroki, Tagami, Miura, & Kato, 2004) to reproductive incompatibility of male individuals with double infections with uninfected females or females infected by a single strain, with each strain individually incompatible with the other, a phenomenon dubbed as cytoplasmic incompatibility (Merçot, Llorente, Jacques, Atlan, & Montchamps‐Moreau, 1995; Perrot‐Minnot et al., 1996).

### 4.2 Elements supporting cytoplasmic incompatibility in the system

Cytoplasmic incompatibility is one of the biological processes that have been studied more intensively for *Wolbachia* (Bourtzis, Braig, & Karr, 2003; Charlat, Bourtzis, & Merçot, 2001; Engelstädter & Telschow, 2009). At present, we know that several strains of *Wolbachia* infect the studied species of *Calligrapha*, and a wealth of indirect evidence points at the existence of incompatibilities between these strains of *Wolbachia*. Unfortunately, proving the existence of cytoplasmic incompatibility in the system would require breeding experiments, which are currently unattainable. One line of reasoning considers the prevalence of double infections in *Calligrapha*. This condition is only stable when the bacteria induce cytoplasmic incompatibility or increase the fitness of the host, with selection favoring in both cases individuals carrying high symbiotic diversity (Vautrin et al., 2007). Additional evidence for cytoplasmic incompatibility clearly emerges when the distribution of each type of *Wolbachia* is examined within each pool of individuals where the conflict should chiefly operate, that is, each single host species. In *C. multipunctata bigsbyana*, the *wCallA* type is widespread and always syntopic (generally as double infections) with the types *wCallB* and *wCallC* (Figure S2a). However, the latter two are allopatric, with *wCallB* distributed from the Pacific coast to the easternmost limit of Lake Ontario, and *wCallC* distributed between this region and the Atlantic shores (Figure S2b). This clear‐cut geographic pattern is suggestive of a tension zone limiting each strain to spread over the range of the other, likely because of cytoplasmic incompatibility as shown for other systems, including other leaf beetles in North America (Roehrdanz & Levine, 2007). Alternatively, this pattern could reflect some kind of environmental filtering for *Wolbachia* (Keller, Windsor, Saucedo, & Werren, 2004), but this explanation is at present more speculative than cytoplasmic incompatibility, and it clashes with the following observations. In particular, the analogous pattern for *C. philadelphica* offers additional indirect evidence for the incompatibility between strains *wCallB* and *wCallC*. As before, *wCallA* occupies the whole range of this species, nearly always coexisting with *wCallB* and *wCallC*, in turn broadly parapatric (Figure S2c). However, the latter coexist in New England and eastern Canada (Figure S2d), but they do in the area of sympatry of two deeply divergent mtDNA lineages of *C. philadelphica*: one group of haplotypes shared (B1) or closely allied (B4, B7) to these in *C. m. bigsbyana* (in the *m*_NL_ lineage), and carrying the *wCallB* type; and one clearly separated from *C. m. bigsbyana* (haplotypes B67–B73 in *p*_EL1_) and ancestrally carrying the *wCallC* type. In this case, incompatibility as a postmating isolation mechanism may be also in the interest of the host retaining the identity of each divergent lineage, and a possible driver for speciation (e.g., Brucker & Bordenstein, 2012; Rokas, 2000; Telschow, Hilgenboecker, Hammerstein, & Werren, 2014; Wade, 2001).

### 4.3 History of *Calligrapha* mtDNA expansion and its association with *Wolbachia*

**Figure 5:**
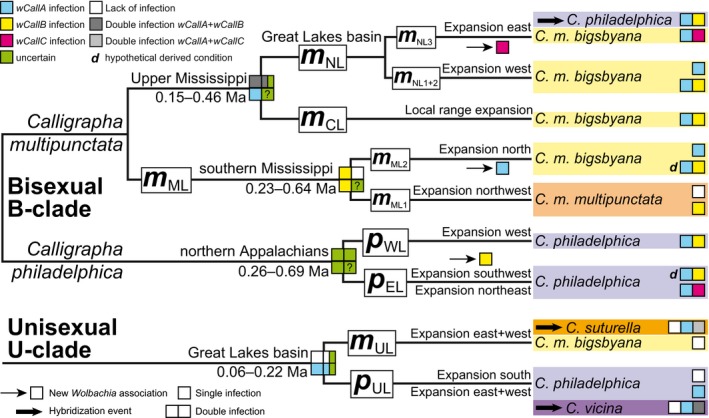
Phylogeographic history of the B‐ and U‐clades of *Calligrapha multipunctata, C. philadelphica*, and their derived unisexual species, *C. suturella* and *C. vicina*, as inferred by Gómez‐Zurita and Cardoso (2019), showing the current association of each lineage with the dominant type of *Wolbachia* infection and the inferred changes in this association through time and space

Gómez‐Zurita and Cardoso (2019) demonstrated that species paraphyly for mtDNA in *Calligrapha* is not at odds with the existence of clear phylogeographic patterns and also coherence with the nuclear genomic background (as represented by the single‐copy gene marker *wingless, Wg*) for individual mtDNA lineages. Several major independent lineages modified and expanded their ranges in different areas of North America in a coherent manner, influenced by glaciation cycles contracting them to a number of refugia from where secondary expansions occurred after the LGM (Gómez‐Zurita & Cardoso, 2019). Interestingly, these lineages have statistically demonstrated specific associations with *Wolbachia* too. Figure 5 condenses the phylogeographic history of these *Calligrapha* lineages, their association with *Wolbachia*, and the inferred changes in this association throughout the history of this group of beetles. The mere existence of the association suggests that *Wolbachia* could have influenced mtDNA evolution in the analyzed species of *Calligrapha*, as proposed in several studies of similar geographic (Baudry, Bartos, Emerson, Whitworth, & Werren, 2003; Raychoudhury et al., 2010) and/or taxonomic scopes (Jäckel, Mora, & Dobler, 2013; Roehrdanz & Levine, 2007). In fact, it was suggested that the occurrence of more or less dramatic symbiont‐driven effects on mtDNA diversity patterns would be the norm when this association exists (Hurst & Jiggins, 2005). However, it is also possible that this association reveals a superimposed combination of ancestral founder events of the endosymbiont, hitchhiking and expanding its own range thanks to the expansion of the beetle populations. Three lines of evidence support that the association may have weak or only local effects on the mtDNA evolution of *Calligrapha*: (a) mtDNA diversity is high and without the signature for selective sweeps or bottlenecks at least for major groups and based on the implementation of Tajima's test of neutrality (Gómez‐Zurita & Cardoso, 2019); (b) mtDNA lineages within each species are also distinctive for a nuclear marker, *wingless*, which reinforces the idea of historical separation of these groups (Gómez‐Zurita & Cardoso, 2019) and not specific selection of mtDNA by endosymbionts; and (c) mtDNA lineages associated with specific sequence types of *Wolbachia*, when expanding their range in an area with a different strain of *Wolbachia*, shift their association, usually incorporating the local strain, independently of their mtDNA type. This could easily occur via paternal transmission from local residents to colonists uninfected or carrying the *wCallA* type only, to account for symbiont incompatibility dynamics, as explained above. The examples below also consider that these shifts could occur via horizontal transmission from the environment or other species infected by the local *Wolbachia* strains without altering the idea of the endosymbiont not affecting mtDNA evolution.

In the case of the B‐clade, *C. philadelphica p*_WL_ and *p*_EL_ lineages originated in the Northern Appalachians during the Middle Pleistocene (Gómez‐Zurita & Cardoso, 2019). Lineage *p*_WL_ spread west, reaching southern Alberta, and it is statistically different from other lineages regarding the type of *Wolbachia* infection. *p*_WL_ individuals are typically characterized by double infections with *wCallA* and *wCallB Wolbachia*, with an ancestor that likely showed this condition already. An evolutionary branch (*p*_EL1_) of linage *p*_EL_ colonized and spread in the northern Appalachians and northeastern Atlantic region, also typically doubly infected with *Wolbachia*, but in this case with *wCallA* and *wCallC* bacteria. The other evolutionary branch (*p*_EL2_) expanded southwest along the Appalachians, reaching the Blue Ridge Mts., accompanied by a derived shift from a *wCallA/wCallC* to *wCallA/wCallB* infection, consistent with the geographic background of the bacteria. This clearly exemplifies the uncoupling of the characteristics of the *Wolbachia* association from the evolution of mtDNA in *Calligrapha*, which shows the history of the host, not a symbiont‐driven mtDNA sweep.

The remaining B‐clade lineages include most of the samples of *C. multipunctata*, with a southern group associated with the Mississippi basin (*m*_ML_), and a northern group distributed from Eastern Canada and New England to the Pacific Northwest (*m*_NL_ and *m*_NC_). The *m*_ML1_ branch of the southern Mississippian lineage is exclusively formed by uninfected individuals or individuals only infected by *wCallB*‐type *Wolbachia*. It is different from its sister lineage, *m*_ML2_, which spread north in the Late Pleistocene and where individuals display either simple infections with *wCallA*‐type *Wolbachia* or double *wCallA/wCallB* infections. It is uncertain whether the ancestor of the *m*_ML2_ lineage carried the double infection of the *wCallA* and *wCallB* types or if it was only host to the former, but the first option received higher probability (56.2%) than the second (32.8%), suggesting a possible early acquisition of the *wCallA* infection during its range expansion north. Derived populations and haplotypes of this mtDNA lineage are mainly distributed around the Great Lakes area and show a generalized double *wCallA/wCallB* infection, statistically different relative to ancestral sources and populations in southernmost locations, with a relative dominance of lack of infection or single *wCallA* infections. The changes in the association with *Wolbachia* in this case would be also congruent with the history and range expansion of the beetles, without noticeable effects of the symbiont on mtDNA diversity or hierarchy.

In the northern lineage of *C. multipunctata*, one group of descendants (*m*_NL2_) remained in the upper courses of the Mississippi and the Missouri Rivers, and they uniformly display a *wCallA/wCallB* association with *Wolbachia*, as other mtDNA lineages in the area. Another group (*m*_NL1_) crossed the Rocky Mountains and arrived to the Pacific Northwest, evolving a different association with *Wolbachia* by losing the *wCallB* type. Contemporarily, another lineage (*m*_NL3_) spread east reaching to Nova Scotia. This evolutionary branch is polymorphic for the type of *Wolbachia* infections, with individuals showing single *wCallA* or double *wCallA/wCallB* infections, as supposedly carried by the ancestor of the lineage. However, most of them show a double *wCallA/wCallC* infection, consistent with the colonization over the area that seems the natural range of the *wCallC* strain of *Wolbachia*. At the edge of this expansion, in Maine and New Brunswick, this *C. multipunctata bigsbyana* lineage hybridized with *C. philadelphica* (Gómez‐Zurita & Cardoso, 2019). Interestingly, the specimens of *C. philadelphica* embedded in this group also inherited with very high probability a double *wCallA/wCallB* infection, the ancestral condition for this whole *C. multipunctata* lineage, and this trait, together with their divergent mtDNA, differentiates them from sympatric *C. philadelphica*, typically with a *wCallA/wCallC* infection. The *m*_CL_ evolutionary branch of the northern *C. multipunctata bigsbyana* lineage colonized the Northern Great Plains and survived the LGM locally. This lineage was ancestrally associated with *wCallA*‐ and *wCallB*‐type *Wolbachia*, coexisting and sharing endosymbiotic makeup with descendants of two divergent mtDNA lineages, as discussed previously, reinforcing the idea of *Calligrapha* changing or adopting the diversity of *Wolbachia* present in the ranges they expand to, without obvious selective sweeps for mtDNA.

As mentioned above, high genetic diversity of the host organelle genome is usually interpreted as *Wolbachia* not having an influence on extant mtDNA diversity. However, it has been shown also analytically that the mere presence of *Wolbachia* in a system already has an impact on mtDNA polymorphism and nonsynonymous substitution rates, among others (Cariou, Duret, & Charlat, 2017). Moreover, there are potential alternative explanations for this pattern without challenging the role of *Wolbachia* in manipulating mtDNA diversity, for example, considering multiple sweeps associated with multiple infections by divergent strains (Frost, Hernández‐Marín, Smith, & Hughes, 2010; Symula et al., 2013). Nevertheless, in the case of *Calligrapha*, the observed pattern is consistent with a dynamics of multiple infection, extinction, or turnover involving few strains of *Wolbachia*, where the strain of bacteria can change, but without evidence for loss or replacement of mtDNA diversity in the host, at least beyond the establishment of the main lineages. The results obtained for *Calligrapha*, where geography or even demography plays a major role in the structure and diversification of mtDNA despite of *Wolbachia*, parallel those obtained for spider mites across China (Chen, Zhang, Du, Jin, & Hong, 2016), or to some extent those for the oak gallwasp in Europe (Rokas, Atkinson, Brown, West, & Stone, 2001), increasing the body of *Wolbachia* literature reporting lack of evidence for selective sweeps, or at least showing that these are not a dominant feature for the mtDNA evolution of the host (Bereczki, Rácz, Varga, & Tóth, 2015; Keller et al., 2004).

### 4.4 Wolbachia is not responsible for current unisexuality in *Calligrapha*

The main motivation of the current study was examining whether reproductive manipulation by *Wolbachia* could explain the deep mtDNA split and the apparent association of one type of mtDNA with unisexuality in *Calligrapha* (Montelongo & Gómez‐Zurita, 2015). The lack of strong evidence for selective mtDNA sweeps in *Calligrapha* already suggests that *Wolbachia* is not manipulating reproduction in this system in ways that condition mtDNA evolution, at least in recent times. These sweeps are only expected to occur when *Wolbachia* alters the outcome of host reproduction inducing unisexuality (Hurst & Jiggins, 2005; Johnstone & Hurst, 1996). Indeed, the lines of *Calligrapha* with the most dramatic alterations in reproduction, that is, those including unisexual taxa in the U‐clade, are either devoid of *Wolbachia* or are infected by the most abundant and widespread strain of *Wolbachia*, which is also present in populations that do not show any reproductive bias.

The history of the U‐clade and the evolution of its association (or lack thereof) with *Wolbachia* reinforce the idea of this association being disconnected from the origin of hybrid unisexual species. In the case of unisexual *Calligrapha* species, *Wolbachia* may at most benefit from this reproductive mode, as has been suggested, for instance, for unisexual *Eusomus* weevils in Europe (Mazur et al., 2016). Taxonomically recognizable unisexual species of *Calligrapha* derive from a female‐only lineage of bisexual *Calligrapha* (Gómez‐Zurita & Cardoso, 2019). The common ancestor of this lineage was inferred in the Great Lakes area during the Late Pleistocene (Gómez‐Zurita & Cardoso, 2019), and with high probability, it was not associated with *Wolbachia*, or only with the *wCallA* strain. This group includes two sublineages, one with *C. multipunctata bigsbyana* and *C. suturella* (*m*_UL_) and one with *C. philadelphica* and *C. vicina* (*p*_UL_), and they also show statistically significant differences in the type of association with *Wolbachia*. The first is typically free from *Wolbachia*, and the second includes a lineage that expanded south, also without *Wolbachia*, and one that spread east and west in the Late Pleistocene and typically with *wCallA Wolbachia*. Despite relatively small samples sizes, both unisexual species embedded in this unisexual group have individuals uninfected, infected by *Wolbachia* of the *wCallA* type, and doubly infected. The latter are with the *wCallB* type in the case of *C. vicina* and the *wCallC* type in the case of *C. suturella*, consistent with the segregated ranges of these *Wolbachia* types, and ruling out any cause/effect association between extant *Wolbachia* and reproductive strategy. Both species originated in the Holocene, and the short evolutionary time since the establishment of these unisexual lineages suggests that their polymorphic association with *Wolbachia* could be the result of independent founder events. This scenario was plausibly considered by Gómez‐Zurita and Cardoso (2019) and would imply multiple origins from maternal parentals with different associations with the bacteria. The evidence amounts to a change in reproductive mode in the analyzed unisexual lineages irrespective of the hypothetical effects of *Wolbachia*, which would not be in principle responsible for this evolutionary transition in *Calligrapha*, as also recognized for other systems (Ma & Schwander, 2017).

### 4.5 Was evidence for the role of *Wolbachia* in the reproductive transition lost?

So far, all available information was interpreted to argue against *Wolbachia* being responsible for the transition from bi‐ to unisexuality in *Calligrapha*. This interpretation considers the relatively strong evidence against mtDNA selective sweeps in this system and the current and inferred ancestral lack of *Wolbachia* or special strain of *Wolbachia* in the unisexual lineage and unisexual species of these beetles. However, there is a particular scenario that would still require the participation of the symbiont for the transition in reproductive mode, yet erasing the required proof for this involvement. This scenario considers that the observed reduced prevalence of *Wolbachia* or trend to single *wCallA* infections of the unisexual mtDNA branch of the *Calligrapha* tree is not the ancestral, but a derived state for this lineage. If unisexual reproduction is a stable condition in this evolutionary lineage of *Calligrapha*, their reproductive independence from males may have relaxed the selective pressures imposed by cytoplasmic incompatibility, which gives advantage to infected females and the spread of superinfections in the populations (Turelli, 1994; Vautrin et al., 2007). This relaxation, together with evolutionary independence from bisexual populations of conspecific *Calligrapha* (in turn potentially reinforced by cytoplasmic incompatibility between superinfected B‐type individuals and uninfected U‐type individuals), could explain the gradual loss of *Wolbachia* in the lineage, as predicted by the theory (Hurst, Jiggins, & Pomiankowski, 2002; Zug et al., 2012). In support to these forces being at play, it is worth noting that there are 14 localities where individuals with B‐ and U‐clade haplotypes are sympatric, often collected on the same plant, and in most of these localities (78.6%), each mtDNA lineage shows a different infection status, whereby the B‐type individuals are usually (79.3%) doubly infected and the U‐type individuals are either uninfected (52.6%) or exclusively infected with *wCallA Wolbachia* (28.9%). These data suggest that horizontal or sexual transmission of *Wolbachia* between these divergent lines is unlikely. In these circumstances, considering the relatively old age of the split between the U and the B mtDNA types, dated at some 2.20–9.46 Ma (Gómez‐Zurita & Cardoso, 2019) and in the same range of spread/extinction dynamics of *Wolbachia* proposed by Bailly‐Bechet et al. (2017), it is possible that the record of the ancestral infection status of the U‐lineage has been lost. With this loss, we may have also lost evidence for the actual liability of *Wolbachia* and of a particular strain not characterized in this study to explain an ancestral sweep (but allowing for subsequent accumulation of mtDNA polymorphism) and transition to unisexuality, responsible for the divergent evolutionary pathways in the first place (see, e.g., Dyer, Burke, & Jaenike, 2011). In this case, the putatively extinct symbiont, which should have been able to induce unisexuality, would have spread in a number of lineages of *Calligrapha* in a wave of multiple hybridization events that also introgressed the taxonomically unascribable U mtDNA type (Montelongo & Gómez‐Zurita, 2015). However, while this possibility is theoretically possible, in the absence of additional evidence, it is safer to dissociate *Wolbachia* from the origin of unisexuality in *Calligrapha*.

## CONFLICT OF INTEREST

The author declares no conflict of interest in relation to the work described in this article.

## AUTHOR CONTRIBUTIONS

JG‐Z designed the study, prepared and analyzed the data, processed and interpreted the results, and wrote the manuscript.

## Data Availability

Vouchers of the specimens used in the study are part of the research collection of the author at the Institute of Evolutionary Biology (CSIC‐Universitat Pompeu Fabra) in Barcelona (Spain). Sequences have been deposited in the public nucleotide sequence repository GenBank under accession numbers LR135794–LR135810.
