# Supplementary material for: Assessment of the role of Wolbachia in mtDNA paraphyly and the evolution of unisexuality in Calligrapha (Coleoptera: Chrysomelidae)
Source: Ecol Evol. 2019 Sep 7;9(19):11198–214. doi: 10.1002/ece3.5621 (PMC6802014; doi:10.1002/ece3.5621)
Supplement: Supplementary file 1 [file ECE3-9-11198-s001.pdf]

SUPPLEMENTARY INFORMATION

Assessment of the role of *Wolbachia* in mtDNA paraphyly and the evolution of unisexuality in *Calligrapha* (Coleoptera: Chrysomelidae)

JESÚS GÓMEZ-ZURITA

Table S1. Samples of *Calligrapha* used for epidemiologic analysis of *Wolbachia*, with information on locality, including georeferences, sample size, mtDNA *cox1* haplotypes (and IBE-JGZ voucher code), and identified MLST of *Wolbachia* and their frequency.

Table S2. *Wolbachia* MLST alleles characterized in the sample of *Calligrapha*. The alleles newly described in this study, arbitrarily assigned letters ***a–e*** to distinguish them, are signaled with an asterisk, and those already reported in the literature are identified with their corresponding number. For each allele, the closest known reference allele is given as well as the number and specific mutations separating them.

Figure S1. Geographic distribution of *Wolbachia* MLSTs characterized from the sample of four species of *Calligrapha*. The maps show the distribution of different sequence types, including: (a) *wCallA1* type; (b) *wCallA2* (blue circles) and *wCallA3*-*wCallA6* (different symbols); (c) *wCallB1* (yellow circles) and localities where *wCallB2–wCallB4* are present; (d) *wCallC1*; and (e) *wCallC2* (fuchsia circles), *wCallC3* (white circles), and localities where *wCallC4*-*wCallC5* are present.

Figure S2. Geographic distribution of *Calligrapha multipunctata* (circles) and *C. philadelphica* (squares) individuals infected by *wCallA* (a, c) and *wCallB* (yellow) and *wCallC* (fuchsia) types of *Wolbachia* (b, d).

Figure S3. Geographic distribution of uninfected individuals of *Calligrapha* (a), individuals infected by a single strain of *Wolbachia* (b), and individuals bearing double *Wolbachia* infections.

Table S1. Samples of *Calligrapha* used for epidemiologic analysis of *Wolbachia*, with information on locality, including georeferences, sample size, mtDNA *cox1* haplotypes (and IBE-JGZ voucher code), and identified MLST of *Wolbachia* and their frequency.

**Taxon**<br>**Locality**	**Latitude**	**Longitude**	**N**	**mtDNA (specimen voucher no.)**	**MLST *Wolbachia (wCall)***
Calligrapha multipunctata bigsbyana (Kirby)					
USA: MI, Schoolcraft Co.	46.0585	-86.2622	1	U5 (C246)	uninfected (1)
Canada: MB, Division no. 15	50.2257	-99.7306	3	U7 (330, 331, 335)	uninfected (1)
Canada: MB, Division no. 15	50.3937	-100.3540	1	B49 (336)	*A1*/*B1* (1)
Canada: MB, Division no. 15	50.4987	-100.9126	3	B13 (329), B16 (338), B49 (337)	*A1*/*B1* (3)
Canada: MB, Division no. 15	50.5803	-101.2332	3	B8 (339), B16 (341), B49 (340)	*A1*/*B1* (3)
Canada: NB, Kent Co.	46.3736	-64.8216	5	B1 (C437), B18 (C435, C436, C438, C439)	*A1*/*C1* (5)
Canada: NB, Madawaska Co.	47.3508	-68.2123	3	B1 (C525, C527), B18 (C526)	*A1*/*C1* (3)
Canada: NB, Northumberland Co.	46.9839	-65.6913	5	B1 (C429), B18 (C431, C432), U8 (C430, C433)	uninfected (2), *A1*/*C1* (3)
Canada: NB, Pictou Co.	45.6589	-62.4200	5	B18 (C393), U8 (C391, C392, C394, C395)	uninfected (4), *A1*/*C1* (1)
Canada: NB, Restigouche Co.	47.6455	-67.3568	6	B1 (C481, C507), B3 (C484), B18 (C480, C482, C483)	*A1*/*C1* (6)
Canada: ON, Haldimand-Norfolk	42.6955	-80.3258	6	B49 (349-351, 358), B51 (352), B52 (357)	*A1*/*B3* (1), *A1*/*B1* (5)
Canada: ON, Ottawa Division	45.3100	-75.6770	8	B1 (CM025-CM027, CM037-CM041)	uninfected (1), *A4* (1), *A1*/*C1* (6)
Canada: ON, Ottawa-Carleton Reg. Mun.	45.3958	-75.5265	1	B1 (CM075)	*A1*/*C1* (1)
Canada: ON, Prescott & Russell United Co.	45.3330	-75.1610	2	B1 (CM053, CM054)	*A1*/*C1* (2)
Canada: ON, Prescott & Russell United Co.	45.5520	-74.4350	4	B1 (CM001-CM004)	uninfected (1), *A1*/*C1* (2), *A4/C1* (1)
Canada: QC, Bonaventure Reg. Co. Mun.	48.2524	-65.9019	5	B1 (C485, C487, C488), B18 (C486, C489)	*A1*/*C1* (5)
Canada: QC, Brome-Missisquoi	45.1890	-72.7480	5	B1 (CM057-CM060, CM108)	uninfected (1), *A1* (2), *A1*/*C1* (2)
Canada: QC, Drummond	45.7978	-72.4591	1	B18 (CM068)	uninfected (1)
Canada: QC, La Côte-de-Beaupré	47.1380	-70.7520	1	B1 (CM055)	*A1*/*C1* (1)
Canada: QC, L'Amiante	46.0570	-71.3550	2	B1 (CM029), B5 (CM028)	*A1*/*C1* (2)
Canada: QC, L'Amiante	45.9359	-71.3772	1	B18 (CM064)	*A1*/*C1* (1)
Canada: QC, Le Haut-Saint-François	45.4840	-71.6780	6	B1 (CM014-CM017, CM022, CM023)	uninfected (2), *A1* (1), *A1*/*C1* (3)
Canada: QC, Le Val-Saint-François	45.5290	-72.0000	1	B1 (CM030)	*A1*/*C1* (1)
Canada: QC, L'Outaouais	45.3828	-75.8170	7	B1 (105, CM089-CM094)	*A1* (1), *A1*/*C1* (6)
Canada: QC, L'Outaouais	45.5143	-76.2350	3	B1 (CM086, CM087), B18 (CM088)	*A1* (1), *A5*/*C1* (1), **B1*/**C1* (1)
Canada: QC, L'Outaouais	45.5290	-76.1802	4	B1 (353-356)	*A1* (1), *A1*/*C1* (3)
Canada: QC, Memphrémagog	45.2530	-72.3390	3	B1 (CM032-CM034)	*A1* (2), *A1*/*C1* (1)
Canada: QC, Nicolet-Yamaska	46.1610	-72.3750	1	B1 (CM056)	*A1*/*C1* (1)
Canada: QC, Roussillon	45.3330	-73.4680	4	B1 (CM049-CM052)	uninfected (1), *A1*/*C1* (3)

Canada: SK, Division no. 7	50.5710	-105.3130	1	B24 (C372)	*A1*/*B1* (1)
Canada: SK, Division no. 11	51.9885	-106.0636	3	B9 (344), B12 (343), B38 (342)	*A1* (2), *A1*/*B1* (1)
USA: AL, Choctaw Co.	31.8227	-88.1796	1	B45 (C315)	*A1* (1)
USA: AL, Escambia Co.	31.0669	-87.0611	3	B39 (C303), B40 (C332), B41 (C333)	*A3* (3)
USA: IA, Clayton Co.	42.7066	-91.0255	7	B49 (C116-C119, C121, C122), B54 (C120)	*A1* (7)
USA: IA, Clayton Co.	43.0436	-91.1868	1	B49 (C190)	*A1* (1)
USA: ID, Bonner Co.	48.4610	-116.8878	4	B1 (C067, C068), B31 (C031, C032)	uninfected (4)
USA: LA, St. Tammany Parish	30.3032	-89.6424	6	B32 (C340), B33 (C339), B46 (C335), B57 (C336, C338), B58 (C334)	uninfected (6)
USA: LA, Washington Co.	30.7618	-89.8313	1	B58 (C317)	uninfected (1)
USA: MA, Middlesex Co.	42.6952	-71.6803	1	U5 (CM112)	uninfected (1)
USA: MA, Worcester Co.	42.4310	-71.8070	1	B18 (CM062)	*A1*/*C1* (1)
USA: ME, Aroostook Co.	46.0225	-67.9103	1	B18 (C503)	*A1*/*C1* (1)
USA: ME, Cumberland Co.	43.6604	-70.3391	3	B18 (CM110), U5 (CM109, CM113)	uninfected (2), *A1*/*C1* (1)
USA: ME, Kennebec Co.	44.3800	-69.7800	1	B18 (CM024)	*A1*/*C1* (1)
USA: ME, Kennebec Co.	44.3999	-69.7745	5	B1 (346), B18 (345, 347, 348), B21 (332)	*A1*/*C1* (5)
USA: ME, Kennebec Co.	44.6348	-69.5024	6	B18 (CM095-CM100)	*A1*/*C1* (6)
USA: ME, Somerset Co.	44.8800	-69.8000	1	B19 (CM048)	*A1*/*C1* (1)
USA: ME, Somerset Co.	45.6370	-70.2540	1	B1 (CM063)	*A1*/*C1* (1)
USA: ME, Washington Co.	45.3220	-67.7197	4	B18 (C470-C473)	*A1*/*C1* (4)
USA: MI, Luce Co.	46.3101	-85.6205	6	B1 (324-328), B6 (323)	uninfected (1), *A1* (1), *A1*/*C3* (2), *A1*/*C1* (2)
USA: MN, Clay Co.	46.8725	-96.4677	8	B9 (C179), B24 (C174, C176), B37 (C172), B49 (C173, C178), B50 (C175), B53 (C177)	*A1*/*B1* (8)
USA: MN, Crawford Co.	43.2793	-91.0482	1	B49 (C168)	*A1*/*B1* (1)
USA: MN, Hubbard Co.	46.8186	-94.7289	4	B10 (C126), B49 (C123), B53 (C124-C125)	*A1*/*B1* (4)
USA: MN, Mille Lacs Co.	45.7508	-93.6708	1	B49 (C171)	*A1*/*B1* (1)
USA: MS, Pearl River Co.	30.6924	-89.8056	1	B57 (C316)	uninfected (1)
USA: ND, Ransom Co.	46.3697	-97.3219	6	B24 (C184-C185, C189), B49 (C186-C187), B53 (C188)	*A1*/*B1* (6)
USA: NH, Coos Co.	44.4770	-71.5750	1	B1 (CM007)	*A1*/*C1* (1)
USA: NH, Grafton Co.	44.1518	-72.0413	7	B1 (CM079-CM080, CM083), B18 (CM081-CM082, CM085), B20 (CM084)	*A1*/*C1* (7)
USA: NH, Hillsborough Co.	42.8479	-71.8848	5	B18 (C406-C410)	*A1* (2), *A1*/*C1* (3)
USA: NH, Rockingham Co.	43.0019	-70.9462	1	B18 (CM111)	uninfected (1)
USA: NY, Albany Co.	42.5182	-74.1461	9	B1 (445, 447-448, 450-452), B2 (453), B18 (446,	uninfected (1), *A1* (2), *A1*/*C1* (6)

				449)	
USA: NY, Clinton Co.	44.5728	-73.4329	3	B1 (C509-C511)	*A1*/*C1* (3)
USA: NY, Grass Island Co.	43.3670	-76.6500	6	B42 (CM018-CM019, CM021, CM035-CM036), B43 (CM020)	*A1*/*B1* (3), *A1*/*B1r* (3)
USA: OR, Clatsop Co.	46.2130	-123.9983	8	B14 (C011-C012, C037-C042)	*A2* (8)
USA: OR, Curry Co.	42.9013	-124.5033	13	B14 (C013, C015-C016, C018, C044-C045, C047-C048), B17 (C014, C017, C043, C046, C049)	*B1* (2), *A2*/*B1* (11)
USA: PA, Jefferson Co.	41.3522	-79.0230	2	B42 (C519), B44 (C520)	*A1*/*B1* (2)
USA: PA, McKean Co.	41.7851	-78.9090	5	B42 (C459-C462), B49 (C458)	*A1*/*B1* (5)
USA: SD, Codington Co.	44.8545	-97.2209	5	B11 (C158, C161), B25 (C159), B49 (C157), B53 (C160)	*A1*/*B1* (5)
USA: SD, Davison Co.	43.7564	-98.0504	1	B53 (C191)	*A1*/*B1* (1)
USA: SD, McCook Co.	43.5911	-97.1914	9	B11 (C146), B24 (C142, C148), B49 (C143), B53 (C144-C145, C149-C150), B55 (C147)	*A1*/*B1* (9)
USA: TX, Bowie Co.	33.5692	-94.4123	9	B46 (C281-C282, C321-C322, C324), B47 (C283-C284, C325), B48 (C323)	uninfected (8), *A1* (1)
USA: VT, Caledonia Co.	44.6000	-72.1800	1	B1 (CM061)	*A1*/*C1* (1)
USA: VT, Orleans Co.	44.7380	-72.1840	4	B1 (CM042-CM045)	*A1* (1), *A1*/*C1* (3)
USA: VT, Orleans Co.	44.8331	-72.1985	2	B18 (CM047), U5 (CM046)	uninfected (1), *A1*/*C1* (1)
USA: VT, Orleans Co.	44.9050	-72.2380	1	B1 (CM006)	*A1*/*C1* (1)
USA: VT, Orleans Co.	44.9090	-72.2230	1	B1 (CM031)	*A1*/*C1* (1)
USA: VT, Orleans Co.	44.9490	-72.1520	3	B1 (CM065-CM067)	uninfected (1), *A1*/*C1* (2)
USA: VT, Orleans Co.	44.9540	-72.1780	6	B1 (CM009-CM013), B18 (CM008)	*A1* (1), *A1*/*C1* (5)
USA: WA, Pacific Co.	46.3304	-124.0615	13	B14 (C001-C010, C350-C352)	uninfected (1), *A2* (12)
USA: WA, Pacific Co.	46.3640	-124.0540	5	B14 (318-320, 322), B15 (321)	uninfected (2), *A2* (3)
USA: WI, Crawford Co.	43.2792	-91.0482	8	B9 (C113, C115), B24 (C109), B49 (C108, C110-C112), B50 (C114)	*A1*/*B1* (8)
USA: WI, Door Co.	44.9453	-87.1854	1	B49 (C139)	*A1*/*B1* (1)
USA: WI, Polk Co.	45.3970	-92.5813	6	B11 (C153), B24 (C155), B49 (C151-C152, C154, C156)	*A1*/*B1* (6)
USA: WV, Randolph Co.	38.4897	-80.0441	9	B49 (CM069-CM074, CM076-CM078)	*A1* (9)
Calligrapha multipunctata multipunctata (Say)					
USA: OR, Baker Co.	44.7451	-117.1688	18	B33 (C024, C027, C029, C057, C060-C061), B34 (C028, C030, C059, C063), B35 (C023, C025, C056, C058, C062), B36 (C054-C055), B56 (C026)	uninfected (2), *B2* (6), *B1* (10)
USA: UT, Cache Co.	41.9739	-111.7640	4	B33 (CM102-CM104, CM107)	*B1* (4)

Calligrapha philadelphica (Linnaeus)					
Canada: AB, Division No. 1	50.0236	-110.6857	2	B27 (C053, C076)	uninfected (1), *A2*/*B1* (1)
Canada: AB, Division No. 1	50.0520	-110.7787	6	B27 (C050-C051, C077-C078, C080-C081)	*A2*/*B1* (6)
Canada: AB, Division No. 11	53.5280	-113.5220	1	B28 (C371)	uninfected (1)
Canada: AB, Division No. 11	53.5380	-113.5529	6	B28 (C033-C034, C072-C075)	uninfected (2), *A2*/*B1* (4)
Canada: AB, Division No. 11	53.5418	-113.5444	4	B28 (C035-C036, C069-C070)	uninfected (1), *A2*/*B1* (3)
Canada: NB, Kent Co.	46.3736	-64.8216	4	B1 (C440-C442), B70 (C443)	*A2*/*B1* (3), *A6*/*C2* (1)
Canada: NB, Madawaska Co.	47.4158	-68.3753	3	B1 (C530), B4 (C528), B72 (C529)	*A2*/*B1* (1), *A1*/*C5* (1), *A1*/*C2* (1)
Canada: NB, Northumberland Co.	46.9839	-65.5913	5	B67 (C424), B68 (C425-C427), B69 (C428)	uninfected (1), *C2* (3), *A6*/*C2* (1)
Canada: NB, Restigouche Co.	47.6455	-67.3568	5	B1 (C474, C476-C478), B73 (C475)	uninfected (1), *A2* (3), *A1*/*B1* (1)
Canada: ON, Lanark Co.	45.2215	-76.1826	1	U11 (389)	*A1* (1)
Canada: ON, Niagara Reg. Municipality	43.1436	-79.3720	4	B59 (391, C090-C091), B60 (C089)	*A1*/*C2* (4)
Canada: ON, Ottawa-Carleton Reg. Mun.	45.4087	-75.7424	12	B29 (283, 390, C082-C088, C099-C101)	uninfected (1), *A1*/*B1* (11)
Canada: ON, South Dundas Co.	45.0718	-75.1500	4	B30 (C373, C386), U13 (C374-C375)	uninfected (1), *A1* (1), *A2*/*B1* (2)
Canada: QC, Bonaventure Reg. Co. Mun.	48.2524	-65.9019	5	B65 (C491-C494), B66 (C490)	*A1*/*C4* (1), *A1*/*C2* (4)
Canada: QC, Les Basques Reg. Co. Mun.	48.0897	-69.1943	2	B59 (C531-C532)	*A1*/*C2* (2)
Canada: QC, L'Outaouais	45.5290	-76.1802	7	B29 (C094-C096, C098), U11 (C093), U12 (392, C097)	*A1* (3), *A1*/*B1* (2), *A1*/*B4* (1), *A5*/*B4* (1)
Canada: QC, Rivière-du-Loup Reg. Co. Mun.	47.8071	-68.8733	5	B59 (C397-C401)	uninfected (2), *C2* (2), *A1*/*C2* (1)
Canada: QC, Témiscouata Reg. Co. Mun.	47.6804	-68.8762	5	B59 (C553-C557)	uninfected (1), *C2* (1), *A1*/*C2* (3)
USA: GA, Morgan Co.	33.5506	-83.2996	1	U1 (C298)	*A1* (1)
USA: ME, Aroostook Co.	46.0225	-67.9103	5	B7 (C498-C499, C501), B67 (C497, C500)	*A2* (1), *A1*/*B1* (1), *A2*/*B1* (1), *A6*/*C2* (2)
USA: ME, Hancock Co.	44.5922	-68.4432	5	B22 (C421), B23 (C417), B26 (C418-C420)	*A1*/*B1* (1), *A2*/*B1* (4)
USA: ME, Kennebec Co.	44.5283	-69.6367	3	B62 (393, 409-410)	*A1*/*C2* (3)
USA: ME, Washington Co.	45.3220	-67.7197	5	B71 (C465-C468, C537)	*A1*/*C2* (1), *A2*/*C2* (4)
USA: MI, Schoolcraft Co.	46.0585	-86.2622	1	U1 (561)	*A1* (1)
USA: MI	-	-	2	U1 (600-601)	uninfected (1), *A1* (1)
USA: MN, Chisago Co.	45.5127	-92.9014	1	U9 (C137)	*A1* (1)
USA: MN, Clay Co.	46.8725	-96.4677	4	U2 (C181), U9 (C180, C182-C183)	uninfected (1), *A1* (3)
USA: MN, Freeborn Co.	43.6285	-93.2998	3	U1 (C131), U10 (C130, C132)	*A1* (3)
USA: NC, Henderson Co.	35.4271	-82.4937	5	U1 (C300, C330-C331), U3 (C301-C302)	uninfected (2), *A1* (1), *A1*/*B1* (2)
USA: NC, Watauga Co.	36.2112	-81.7759	7	B64 (C310), U1 (C307-C309, C311-C313)	*A1*/*B1* (5), *A5*/*B4* (2)
USA: NH, Grafton Co.	44.1518	-72.0413	2	U1 (388, C092)	uninfected (1), *A1*/*C2* (1)
USA: NY, Clinton Co.	44.5728	-73.4329	5	B59 (C513-C514, C516-C517), U11 (C515)	*A1* (1), *A1*/*C2* (4)

USA: NY, Clinton Co.	44.7778	-73.3772	2	B61 (C405), U1 (C403)	uninfected (1), *A1*/*C2* (1)
USA: NY, Essex Co.	44.3662	-73.8428	1	U11 (C504)	*A1* (1)
USA: NY, Essex Co.	44.4242	-73.9197	2	B59 (C495-C496)	*A1*/*C2* (2)
USA: OH, Pike Co.	38.8948	-82.5967	1	U1 (117)	uninfected (1)
USA: PA, Jefferson Co.	41.3522	-79.0230	2	B63 (C524), U1 (C522)	uninfected (1), *A1*/*B1* (1)
USA: PA, McKean Co.	41.7704	-78.8816	5	U1 (C445-C448), U4 (C449)	uninfected (4), *A1* (1)
USA: PA, York Co.	40.0724	-76.8975	5	B63 (C453-C454, C456-C457), U1 (C455)	uninfected (1), *A1* (3), *A1*/*A6* (1)
Calligrapha suturella Schaeffer					
Canada: QC, Comunauté-Urbaine-de-Québec	46.9328	-71.3669	1	U5 (129)	*A1*/*C1* (1)
Canada: QC, L'Amiante	45.9667	-71.3500	1	U5 (128)	uninfected (1)
USA: ME, Cumberland Co.	43.6604	-70.3391	1	U5 (C103)	uninfected (1)
USA: ME, Kennebec Co.	44.3999	-69.7745	2	U5 (333, 555)	uninfected (1), *A1* (1)
USA: ME, Somerset Co.	45.6500	-70.2500	1	U6 (130)	*A1*/*C1* (1)
USA: MI, Ogemaw Co.	44.2197	-84.2214	1	U5 (334)	uninfected (1)
Calligrapha vicina Schaeffer					
Canada: South Dundas Co.	45.0718	-75.1500	2	U3 (C376, C385)	*A1* (2)
USA: NY, Clinton Co.	44.5728	-73.4329	1	U3 (C512)	uninfected (1)
USA: NY, Clinton Co.	44.7778	-73.3772	2	U3 (C402, C404)	uninfected (2)
USA: PA, Jefferson Co.	41.3522	-79.0230	1	U3 (C523)	*A1*/*B1* (1)
USA: PA, McKean Co.	41.7851	-78.9090	1	U3 (C463)	uninfected (1)

Table S2. *Wolbachia* MLST alleles characterized in the sample of *Calligrapha*. The alleles newly described in this study, arbitrarily assigned letters ***a–e*** to distinguish them, are signaled with an asterisk, and those already reported in the literature are identified with their corresponding number. For each allele, the closest known reference allele is given as well as the number and specific mutations separating them.

Locus	Allele	GenBank Acc. No.	Ref. allele	No. diff.	Allele differences[a]
gatB	***a****	LR135794	238[b]	5	$G_{54}A$, $T_{130}C$, $G_{147}A$, $A_{207}G$, $A_{291}G$
	b*	LR135795	238[b]	6	(as before +) $G_{106}A$
	c*	LR135796	238[c]	10	$G_{54}A$, $A_{226}G$, $A_{291}G$, $T_{318}C$, $A_{321}G$, $C_{324}T$, $T_{339}C$, $A_{342}G$, $T_{348}A$, $A_{369}G$
	d*	LR135797	32	1	$A_{175}G$
coxA	***a*** = 33	LR135798	-	-	-
	b*	LR135799	33	1	$G_{394}A$
hcpA	***a*** = 42	LR135800	-	-	-
	b*	LR135801	42	1	$A_{311}G$
	c*	LR135802	42	1	$A_{383}C$
ftsZ	***a*** = 32	LR135803	-	-	-
	b*	LR135804	32	1	$G_{76}A$
	c = 75	LR135805	-	-	-
fbpA	***a*** = 36	LR135806	-	-	-
	b*	LR135807	36	1	$G_{361}A$
	c*	LR135808	36	4	$G_{366}A$, $A_{379}G$, $G_{382}A$, $A_{426}G$
	d*	LR135809	36	1	$A_{50}G$
	e*	LR135810	36	1	$G_{105}C$

[a]Information shown as R_xN, indicating the character state R in position x of the reference sequence and the observed character state N in the new allele.
[b]Alleles ***a*** and ***b*** are only one ($G_{170}A$) and two mutations ($A_{106}G$ and $G_{170}A$) away, respectively, from allele N5 (KP265899) characterized from *Andrena sp.* 2 (Gerth et al. 2015).
[c]Allele ***c*** is eight mutations ($G_{125}A$, $C_{126}T$, $T_{172}C$, $G_{226}A$, $G_{231}A$, $T_{264}C$, $G_{294}A$ and $T_{297}C$) away from allele N3 (KP798253) characterized from *Nomada conjugens* (Gerth et al. 2015).

Gerth M, Saeed A, White JA, Bleidorn C (2015). Extensive screen for bacterial endosymbionts reveals taxon-specific distribution patterns among bees (Hymenoptera, Anthophila). *FEMS Microbiol Ecol* **91**: fiv047.

SUPPLEMENTARY INFORMATION

Assessment of the role of *Wolbachia* in mtDNA paraphyly and the evolution of unisexuality in *Calligrapha* (Coleoptera: Chrysomelidae)

JESÚS GÓMEZ-ZURITA

Figure S1. Geographic distribution of *Wolbachia* MLSTs characterized from the sample of four species of *Calligrapha*. The maps show the distribution of different sequence types, including: (a) *wCallA1* type; (b) *wCallA2* (blue circles) and *wCallA3*-*wCallA6* (different symbols); (c) *wCallB1* (yellow circles) and localities where *wCallB2–wCallB4* are present; (d) *wCallC1*; and (e) *wCallC2* (fuchsia circles), *wCallC3* (white circles), and localities where *wCallC4*-*wCallC5* are present.

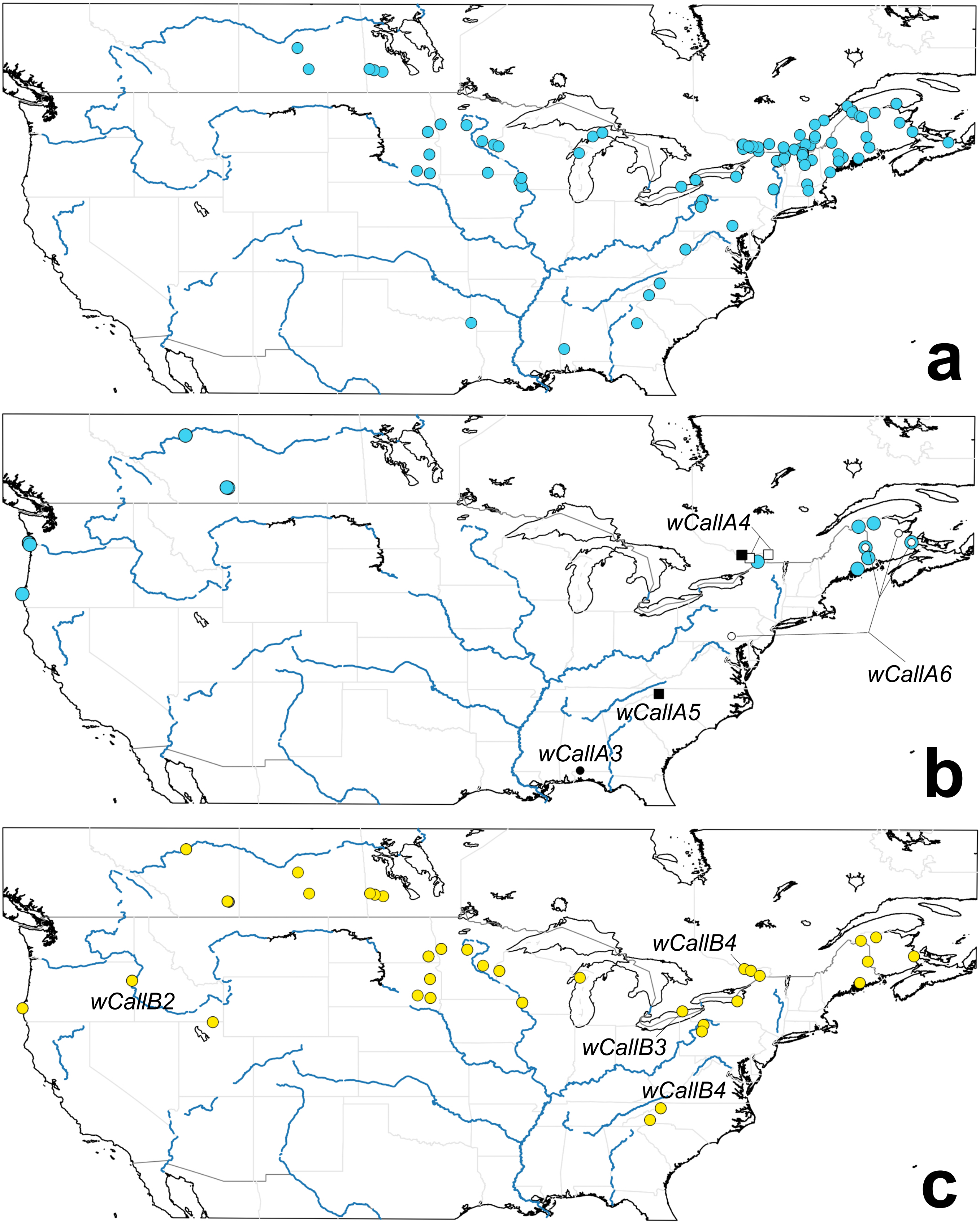
a
wCallA4
wCallA6
wCallA5
wCallA3
b
wCallB4
wCallB2
wCallB3
wCallB4
c

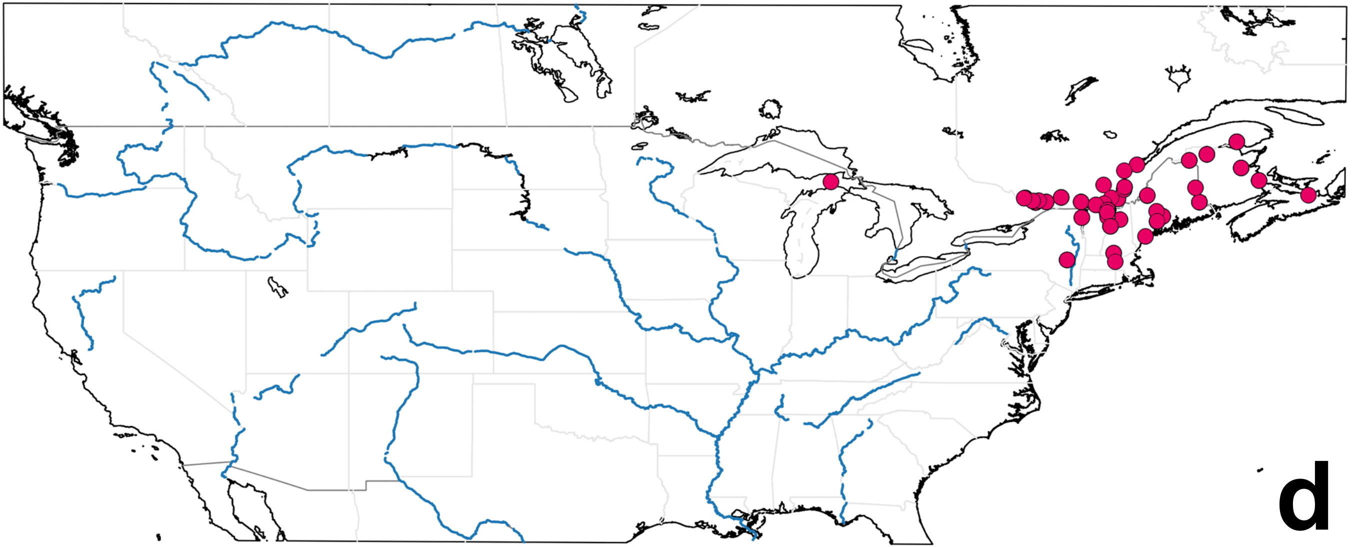
d

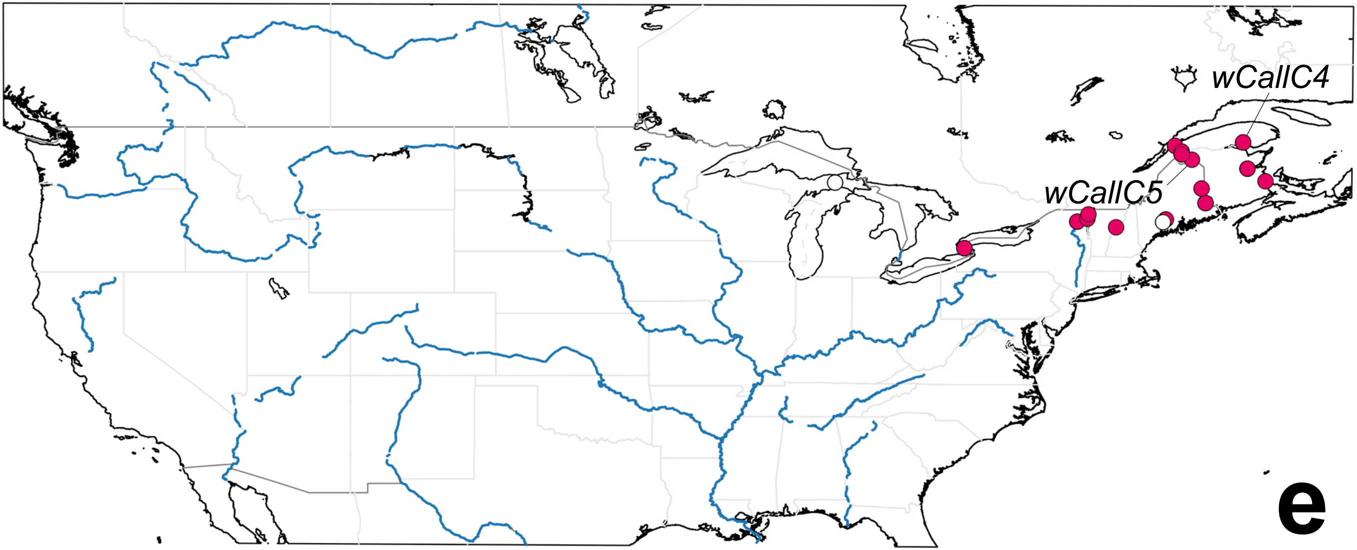
wCallC4
wCallC5
e

SUPPLEMENTARY INFORMATION

Assessment of the role of *Wolbachia* in mtDNA paraphyly and the evolution of unisexuality in *Calligrapha* (Coleoptera: Chrysomelidae)

JESÚS GÓMEZ-ZURITA

Figure S2. Geographic distribution of *Calligrapha multipunctata* (circles) and *C. philadelphica* (squares) individuals infected by *wCallA* (a, c) and *wCallB* (yellow) and *wCallC* (fuchsia) types of *Wolbachia* (b, d).

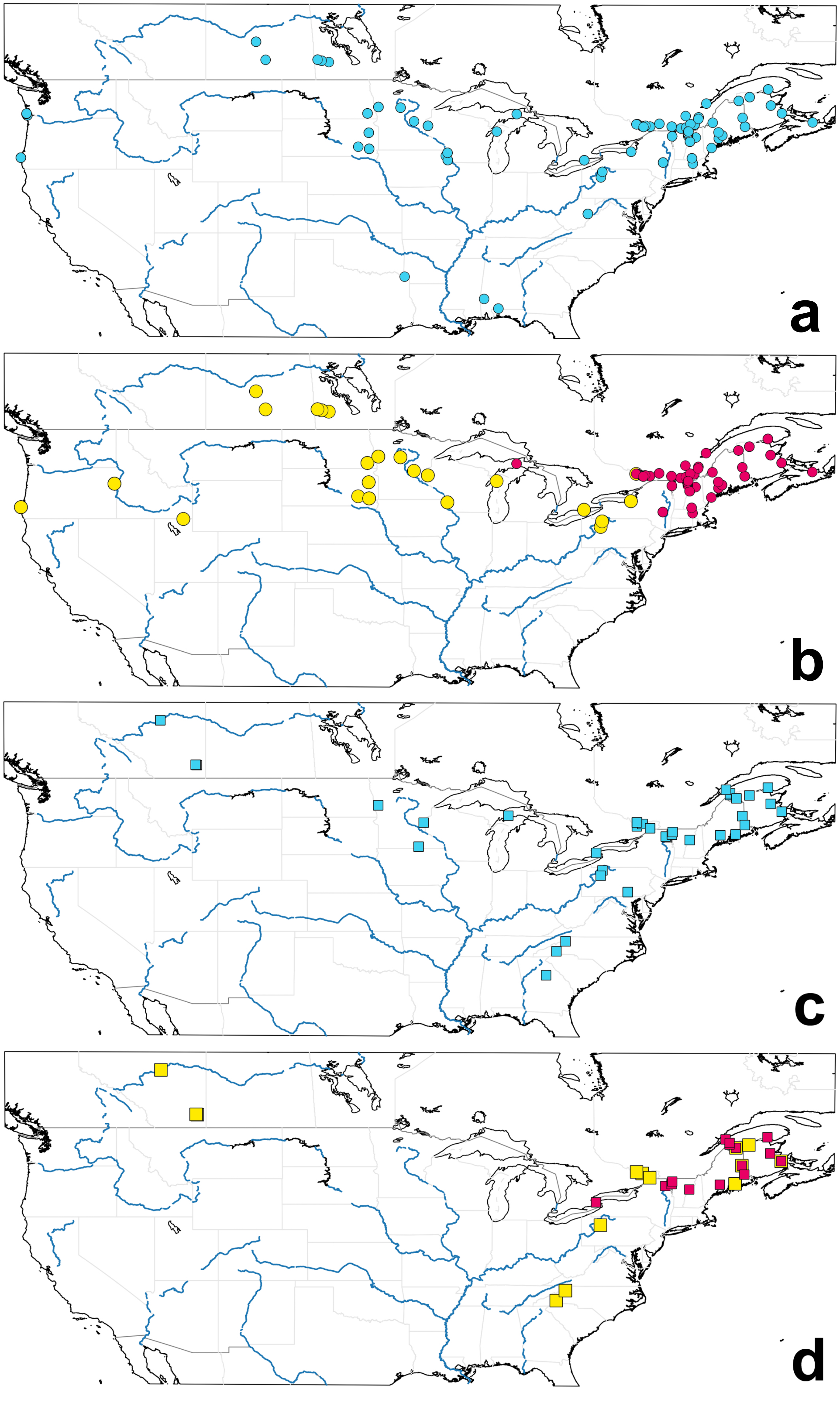
a
b
c
d

SUPPLEMENTARY INFORMATION

Assessment of the role of *Wolbachia* in mtDNA paraphyly and the evolution of unisexuality in *Calligrapha* (Coleoptera: Chrysomelidae)

JESÚS GÓMEZ-ZURITA

Figure S3. Geographic distribution of uninfected individuals of *Calligrapha* (a), individuals infected by a single strain of *Wolbachia* (b), and individuals bearing double *Wolbachia* infections.

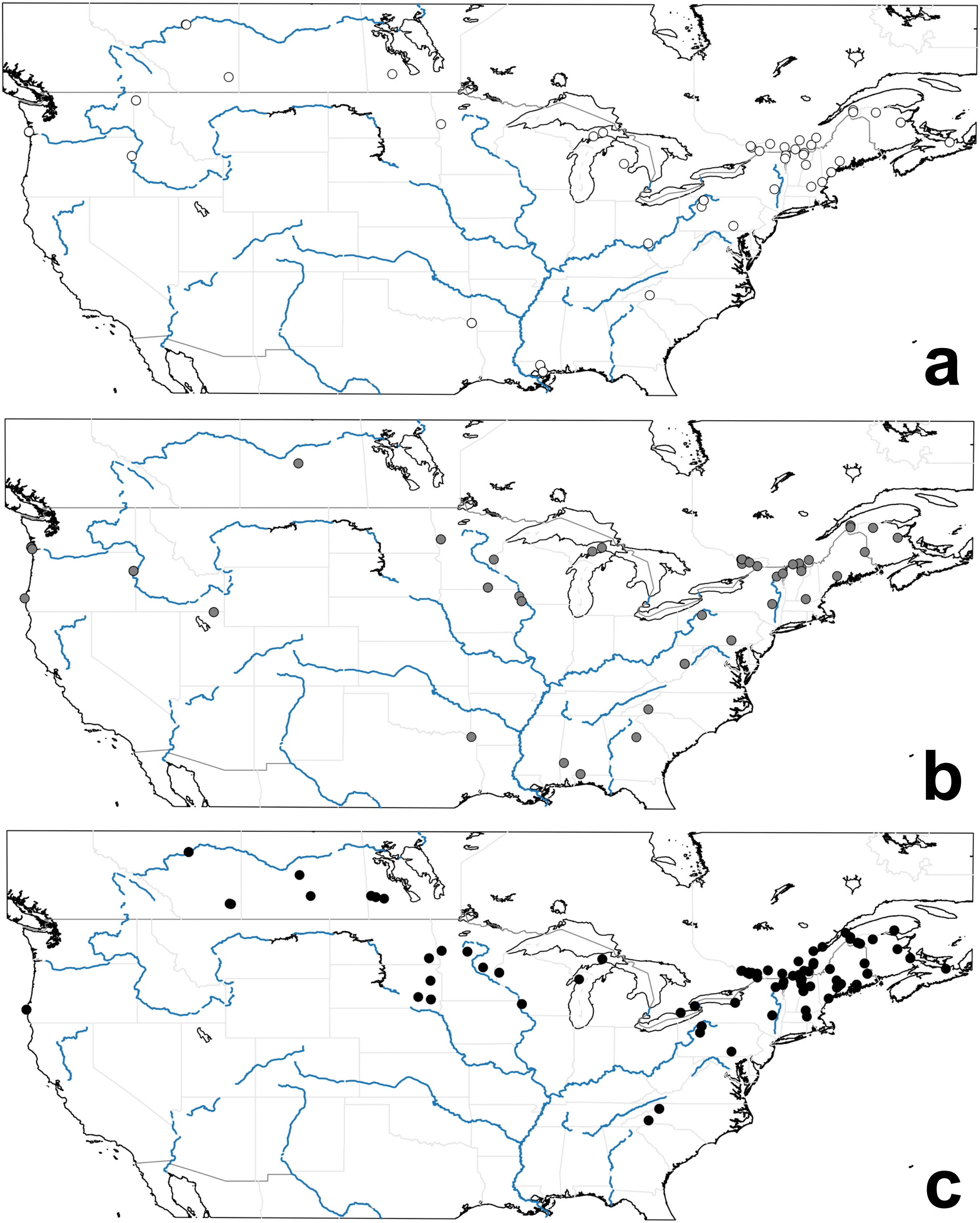
a
b
c
